# Impaired angiogenesis and tumor development by inhibition of the mitotic kinesin Eg5

**DOI:** 10.18632/oncotarget.1490

**Published:** 2013-10-26

**Authors:** Prisca Exertier, Sophie Javerzat, Baigang Wang, Mélanie Franco, John Herbert, Natalia Platonova, Marie Winandy, Nadège Pujol, Olivier Nivelles, Sandra Ormenese, Virginie Godard, Jürgen Becker, Roy Bicknell, Raphael Pineau, Jörg Wilting, Andreas Bikfalvi, Martin Hagedorn

**Affiliations:** ^1^ Univ. Bordeaux, LAMC, UMR 1029, F-33405 Talence, France; ^2^ INSERM, LAMC, UMR 1029, F-33405 Talence, France; ^3^ Ruhr-Universität Bochum, Medizinische Fakultät; Abt. f. Anatomie und Embryologie, D-44780 Bochum, Germany; ^4^ Molecular Angiogenesis Group, Institute of Biomedical Research, Univ Birmingham, Medical School, Edgbaston, Birmingham, UK; ^5^ GIGA, Zebrafish Facility, Tour B34, Université de Liège, Belgium; ^6^ GIGA, Unité de Biologie Moléculaire et Génie Génétique, Tour B34, Université de Liège, Belgium; ^7^ GIGA, Imaging and Flow Cytometry Facility, Tour B34, Université de Liege, Belgium; ^8^ Zentrum Anatomie, Abteilung Anatomie und Zellbiologie, Georg-August-Universität Goettingen, Germany; ^9^ Animalerie mutualisée, University of Bordeaux I, Talence, France

**Keywords:** Angiogenesis, Eg5 kinesin, Mklp2 kinesin, VEGF, ispinesib

## Abstract

Kinesin motor proteins exert essential cellular functions in all eukaryotes. They control mitosis, migration and intracellular transport through interaction with microtubules. Small molecule inhibitors of the mitotic kinesin KiF11/Eg5 are a promising new class of anti-neoplastic agents currently evaluated in clinical cancer trials for solid tumors and hematological malignancies. Here we report induction of Eg5 and four other mitotic kinesins including KIF20A/Mklp2 upon stimulation of in *vivo* angiogenesis with vascular endothelial growth factor-A (VEGF-A). Expression analyses indicate up-regulation of several kinesin-encoding genes predominantly in lymphoblasts and endothelial cells. Chemical blockade of Eg5 inhibits endothelial cell proliferation and migration in *vitro*. Mitosis-independent vascular outgrowth in aortic ring cultures is strongly impaired after Eg5 or Mklp2 protein inhibition. *In vivo*, interfering with KIF11/Eg5 function causes developmental and vascular defects in zebrafish and chick embryos and potent inhibition of tumor angiogenesis in experimental tumor models. Besides blocking tumor cell proliferation, impairing endothelial function is a novel mechanism of action of kinesin inhibitors.

## INTRODUCTION

The ability of solid tumors to attract blood vessels (tumor angiogenesis) is one of the rate-limiting steps for tumor progression [1]. Vascular endothelial growth factor (VEGF-A) is a key hypoxia-induced angiogenic protein secreted by tumor cells acting on the endothelium to induce and sustain new blood vessel growth [2]. Neutralizing VEGF-A with drugs such as the humanized anti-VEGF-A antibody bevacizumab (Avastin) potently blocks tumor growth in numerous animal models. Associated with standard chemotherapy, bevacizumab is used worldwide against multiple cancer types and allows prolonged or progression-free survival. However, by far not all patients respond to anti-VEGF therapy and severe side effects such as hypertension and proteinuria have been reported, a fact which has led to the retirement of bevacizumab as a treatment for metastatic breast cancer [3]. It is therefore a challenge to explore the molecular networks that regulate blood vessel growth to identify novel druggable targets.

To discover novel downstream effectors of VEGF-A activity in the endothelium *in vivo*, we monitored global gene expression changes after application of recombinant human VEGF-A on the differentiated day-13 chick chorio-allantoic membrane (CAM) [4]. Numerous known genes associated with angiogenesis were up regulated by VEGF-A. Among the new ones, KIF4A, KIF11/Eg5, KIF15, KIF20A/Mklp2 and KIF23, all genes encoding mitotic kinesins, were consistently up regulated.

Kinesins make up a family of about 45 proteins in humans; at least 12 of them are implicated in mitosis [5]. KIF11 encodes the Eg5 protein, which is essential for cell division [6]. Murine Knsl1 (KIF11) null-embryos die prior to implantation between morula and blastocyst stage [7, 8]. KIF20A/Mklp2 exerts important functions during mitosis by ensuring cleavage furrow formation and cytokinesis [9].

First evidence that kinesin inhibition might be explored as a new anti-cancer strategy came in 2004 [10] and chemical inhibitors of Eg5 have been designed and tested with success against solid tumors in preclinical tumor models [11]. Consequently, there are increasing numbers of clinical trials investigating the efficacy of Eg5 inhibitors alone or associated with classical chemotherapy in hematological and solid malignancies [12]. There is a general consent that inhibitors of the mitotic kinesins, especially Eg5, have the potential to overcome side-effects associated with classical microtubule targeting agents such as Taxol, which include neutropenia, hair loss and peripheral neuropathy as well as resistance, thereby often limiting their usability [5, 13].

Mitotic kinesins have been studied in the context of cell division almost exclusively in tumor cells [12]. Beside their role in mitosis, an increasing number of reports point to the possibility that they may exert other biological functions. KIF11/Eg5 plays an important role in normal and cancer cell migration [14, 15]. Only recently, an important role of Eg5 in protein translation has been discovered [16] and KIF4A has been shown to be implicated in neuronal survival [17]. Since KIF11/Eg5 and KIF20A/Mklp2 are promising drug targets, we sought to investigate the impact of their respective inhibitors on angiogenesis, a process that is central to tumor progression.

## RESULTS

### VEGF-A-induced gene expression in vivo

**Figure 1:**
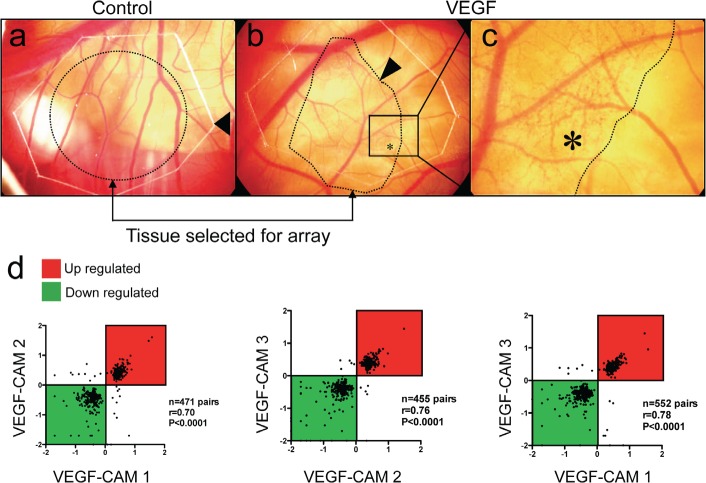
Affymetrix GeneChip screening for VEGF-A-induced genes during CAM vascularization Solvent or human recombinant VEGF-A was deposited on the differentiated CAM at day 13 of development. (a, b, c) 24h later, tissue with visible newly formed capillaries (asterisk in c) was isolated and further processed for mRNA isolation (n=3 CAMs per group). Control CAMs (a) did not show any vascular alterations (arrow: border of the carrier plastic disc). (d) To verify reproducibility of these experiments, fold-change values of genes regulated in VEGF-treated CAMs were plotted against each other for correlation analysis. Significant correlations (Spearmans r ranging from 0.7 to 0.78, P<0.0001) were found for all comparisons indicating that VEGF effects on the CAM were consistent.

Human recombinant VEGF-A induces growth of new capillaries inside the CAM within 24h (Fig. 1b, c). Chicken microarray analysis of angiogenic areas of three individual CAMs was performed. 317 probes showed significantly increased expression (more than 2-fold in at least two out of three comparisons; Table S1). Fold-changes of significantly regulated genes in VEGF-A-stimulated CAMs replicates showed strong correlation (Spearman R ranging from 0.7 to 0.78, P<0.0001 in all comparisons: VEGF CAM 1 vs. VEGF CAM 2, VEGF CAM2 vs. VEGF CAM 3, VEGF CAM 1 vs. VEGF CAM 3; Fig. 1d).

An automated reciprocal blast search identified human orthologs of the 317 chick genes and an *in silico* prediction of endothelial vs. non-endothelial expression of these revealed those with expression enriched in endothelium [19, 25]. 206 human ortholog genes were identified and submitted to gene ontology (GO) using the DAVID interface (Table S2). Interestingly, GO terms associated with mitosis were highly significantly enriched (11.6-fold, FDR: 7.44×10^−13^). Another annotation cluster identified GO term “blood vessel development” (enrichment 5.5-fold, FDR 1.66×10^−4^). A total of 53 single genes with human orthologs and preferential endothelial expression (q-value >0.5) were identified (Table 1). This list contained numerous key angiogenic regulators with known endothelial expression indicating that relevant biological material was isolated for microarray analysis.

**Table 1:** Vascular gene expression program induced by VEGF-A

Human gene	Gene product	VEGF vs Control (mean)	q-value	Ratio ‰ EC-EST/Non-EC-EST
KIF11	kinesin family member 11	23.22	0.10	--
CENPL	centromere protein L isoform 2	4.56	0.29	--
TEK	TEK tyrosine kinase, endothelial precursor	4.39	0.01	--
CENPE	centromere protein E	4.05	0.29	--
ECSM2	hypothetical protein LOC641700	3.45	0.00	--
CD34	CD34 antigen isoform a	2.96	0.00	--
CLDN5	claudin 5	2.88	0.29	--
DPY19L1	dpy-19-like 1	2.65	0.02	--
MYCT1	myc target 1	2.59	0.00	--
ZNF521	zinc finger protein 521	2.59	0.00	--
CDH5	cadherin 5, type 2 preproprotein	2.54	0.00	--
SOX18	SRY-box 18	2.48	0.29	--
C13orf3	hypothetical protein LOC221150	2.28	0.29	--
SOX17	SRY-box 17	2.28	0.10	--
LMO2	LIM domain only 2	2.20	0.29	--
LIFR	leukemia inhibitory factor receptor precursor	2.08	0.00	--
PECAM1	platelet/endothelial cell adhesion molecule (CD31 antigen)	2.86	0.00	171.25
SEC14L1	SEC14 (S. cerevisiae)-like 1 isoform a	2.60	0.00	61.47
TIE1	tyrosine kinase with immunoglobulin-like and EGF-like domains 1	4.20	0.00	35.13
PDGFB	platelet-derived growth factor beta isoform 1, preproprotein	2.21	0.01	21.96
PRCP	prolylcarboxypeptidase isoform 1 preproprotein	6.37	0.00	17.56
RASGRP3	RAS guanyl releasing protein 3 (calcium and DAG-regulated)	2.97	0.04	17.56
PODXL	podocalyxin-like isoform 2 precursor	2.27	0.00	13.17
NRP1	neuropilin 1 isoform a	2.42	0.00	12.55
MYO1C	myosin IC isoform c	3.25	0.00	11.86
ELK3	ELK3 protein	2.07	0.01	10.25
LAMA4	laminin, alpha 4 isoform 1 precursor	2.68	0.00	9.27
KDR	kinase insert domain receptor (a type III receptor tyrosine kinase)	3.19	0.29	8.78
CDH13	cadherin 13 preproprotein	2.41	0.10	8.78
IFNGR1	interferon gamma receptor 1 precursor	2.05	0.29	8.78
USP1	ubiquitin specific protease 1	3.29	0.26	6.59
SEC24C	SEC24-related protein C	2.71	0.05	6.59
C14orf108	chromosome 14 open reading frame 108	2.20	0.05	6.59
SERPINH1	serine (or cysteine) proteinase inhibitor, clade H, member 1 precursor	2.24	0.00	6.23
KIAA1671	KIAA1671 protein	2.41	0.18	5.85
FLI1	Friend leukemia virus integration 1	2.75	0.32	4.39
MKI67	antigen identified by monoclonal antibody Ki-67	2.55	0.26	4.39
BUB1	BUB1 budding uninhibited by benzimidazoles 1 homolog	2.50	0.44	4.39
TM4SF18	transmembrane 4 L six family member 18	2.34	0.44	4.39
NUSAP1	nucleolar and spindle associated protein 1 isoform 2	3.06	0.12	3.84
KIF20A	kinesin family member 20A	3.66	0.26	3.66
DOCK9	dedicator of cytokinesis 9	4.73	0.30	3.51
AKAP12	A-kinase anchor protein 12 isoform 2	2.62	0.25	3.29
RRM1	ribonucleoside-diphosphate reductase M1 chain	2.60	0.07	3.22
PDE4B	phosphodiesterase 4B, cAMP-specific isoform 2	3.89	0.29	3.14
LMBR1	limb region 1 protein	2.43	0.29	3.14
PSMD1	proteasome 26S non-ATPase subunit 1	2.91	0.02	3.05
				
CLIC2	chloride intracellular channel 2	2.43	0.29	2.93
PRPF8	U5 snRNP-specific protein	2.10	0.07	2.85
NUP93	nucleoporin 93kDa	2.84	0.26	2.79
RBBP4	retinoblastoma binding protein 4	1.85	0.29	2.56
AMD1	S-adenosylmethionine decarboxylase 1 isoform 1 precursor	2.08	0.46	2.51
GOT2	aspartate aminotransferase 2 precursor	2.50	0.02	2.45

In silico endothelial-enriched ortholog genes (q-value <0.5) regulated more than 2-fold in at least 2 of 3 comparisons are shown (n=53). As expected, and consistent with active vascular network formation, numerous key angiogenic regulators were induced, such as TEK, CD34, SOX18, LMO2, PECAM1, NRP1, FLI1 and KDR (italicized). Also note induction of mitotic kinesins, KIF11 (encoding Eg5) and KIF20A (in bold). 317 probe sets with significant increase after VEGF-A stimulation in at least two out of three comparisons are shown in Supplemental Table S1.

One of the genes with strong up-regulation and specific prediction of endothelial cell over-expression was KIF11 (coding for the Eg5 protein), a molecule that has not yet been studied in the context of angiogenesis. Up-regulation of a total of five kinesins by VEGF-A was confirmed by semi-quantitative qPCR and was 4.79 for KIF4A, 3.8 for KIF11, 6.25 for KIF15, 5.58 for KIF20A and 2.95 for KIF23 using HNRPH1 as normalizer because its expression did not differ between control and VEGF-treated CAMs (Supplemental Table S1, last line). Increased production of Eg5 protein was further confirmed *in vitro*, after stimulating HUVECs with VEGF_165_ protein. Increase in Eg5 protein could be abolished by simultaneous application of the neutralizing anti-VEGF antibody Avastin (Fig. S1).

### Kinesin expression and localization

We performed an *in silico* bioninformatic screen [25] on all genes encoding kinesins. Eight out of 38 kinesin transcripts showed enrichment in the endothelial EST pool more than two-fold, including the VEGF-A-induced kinesins KIF11, KIF15 and KIF20A (Table S3).

**Figure 2:**
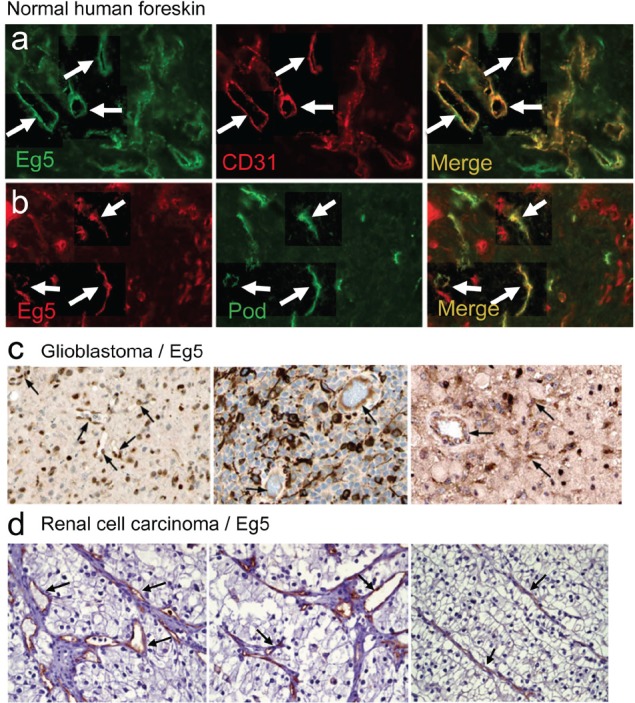
Eg5/KIF11 expression studies in normal and cancerous tissues

In freshly isolated human foreskin, Eg5 staining overlapped to a great extent with that of CD31, indicating that blood endothelial cells strongly express Eg5 protein (Fig. 2a). Lymphatic vessels identified by podoplanin immunoreactivity were also Eg5 positive (Fig. 2b). Immunohistological analysis of human glioblastoma samples revealed staining of Eg5 in endothelial and tumor cells (Fig. 2c). In renal cell carcinoma, predominant staining was observed in capillaries (Fig. 2d). Mklp2/KIF20A protein showed an even stronger expression in endothelial cells in several normal tissues (heart, placenta, endometrium, oral mucosa; Fig. S4a-d) and glioblastoma vessels (Fig. S4e, f). KIF20A transcripts were found mostly in ECs in glioblastoma (n=4 patients, arrows; Fig. S4h), matching the vascular localization of CD31 (Fig. S4g). All five VEGF-induced kinesins are over expressed in a large number of human malignancies as evidenced by Oncomine analysis (Fig. S2a). In small cell lung cancer, fibrosarcoma and glioblastoma, these kinesins are found up regulated, compared to normal tissue (Fig S2b-d). We further focused on KIF11 and KIF20A expression in glioblastoma and found general over-expression in this pathology in two additional studies (Fig S3, left graphs). When expression values were plotted individually per patient, a heterogeneous expression pattern was revealed with about one third of patients under- expressing KIF11 and KIF20A, whereas two-thirds of patients over-express both genes (Fig S3, right graphs).

(a, b) Sections of human foreskin were double-stained with anti-Eg5 and either anti-CD31 or anti-podoplanin antibodies. CD31-positive blood capillaries were also positive for Eg5 (arrows). (b) Colocalisation of Eg5 and podoplanin was also observed in lymphatic vessels (arrows). (c) Immunohistological staining reveals also expression of Eg5 in tumor blood vessels in three different glioblastoma patients (arrows). (d) Strong vascular expression for Eg5 is also found in renal cell carcinoma patients (arrows).

To further shed light on the co-expression of the five kinesins, we performed co-expression analysis using KIF11 gene as bait (Fig. S5a). Expression was low in normal brain, but elevated in anaplastic oligodendroglioma (French_brain study^1^) and all five kinesins were highly co-expressed (correlation from 0.903 for KIF4A to 0.872 for KIF15; black arrows). Similar co-expression could be evidenced in a glioblastoma study (Freje_brain study; Fig. S5b). Co-expression could be linked to other pathological features such as the vascularization state of a tumor, as evidenced in the Wurmbach_liver study for hepatocellular carcinoma: KIF11, KIF4A and KIF15 were strongly co-expressed with KIF20A (>0.8; Fig. S6) and expression levels increased with the degree of vascularization and were highest in tumors with macroscopic vascular invasion.

1Oncomine nomenclature to identify original studies. Detailed reference can be found at www.oncomine.org.

We also used data provided by the BioGPS project [26] to compare KIF11 and KIF20A expression in 84 human tissues and cell lines. Only 8 cell lines had relative KIF11 mRNA levels higher than 20 (Fig. S7), highest levels were found in 721_B_lymphoblasts (274.8), followed by other lymphoblastic lines and CD105^+^ and CD34^+^ endothelial cells (167.95 and 64.75). Highly comparable data were found for KIF20A, relative expression values of KIF11 and KIF20A were highly correlated (Spearman r=0.8492, P<0.0001). These expression data point to a potential role of kinesins in the angiogenic process.

### Eg5 blockade inhibits proliferation of endothelial cells and tumor cell lines

**Figure 3:**
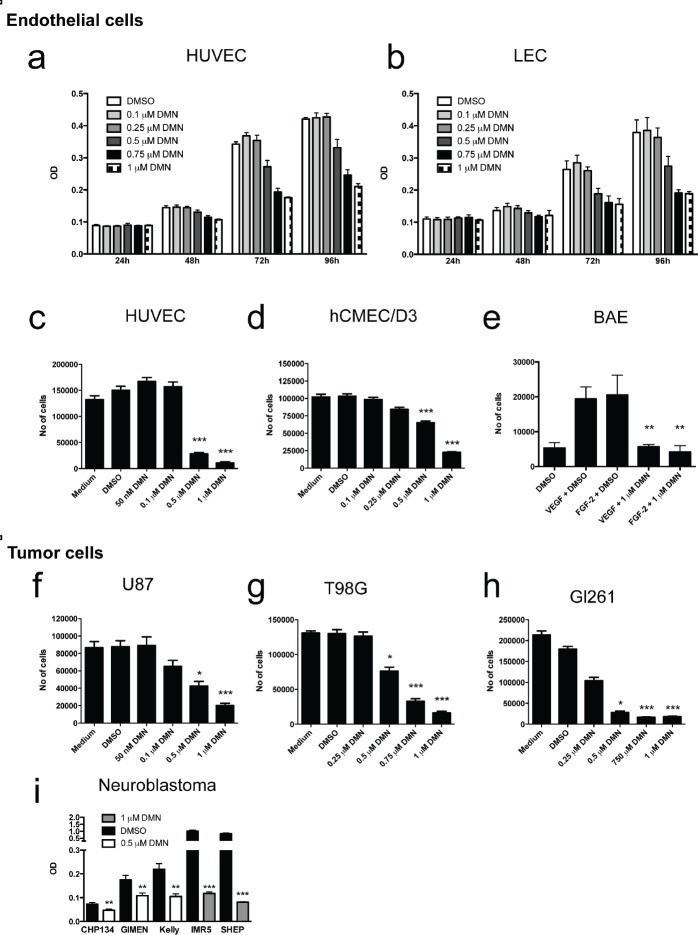
Chemical inhibition of Eg5 in normal and malignant cells

Growth of HUVECs and LECs was inhibited by dimethylenastron (DMN) in a dose- and time-dependent manner (Fig. 3a-c). Inhibition became evident after 72h of treatment with first effects at 0.5 μM and maximal effects at 1 μM. Significant growth inhibition at doses from 0.5 to 1 μM was also observed in the brain microcapillary endothelial cell line hCMEC/D3 (Fig. 3d), and bovine aortic endothelial cells (BAE) stimulated with VEGF-A or FGF-2 in reduced growth medium (Fig. 3e). Eg5 blockade at 1 μM led to complete growth inhibition after 72h, regardless of the type of mitogen used (Fig. 3i). Similar inhibition was observed in the human malignant glioma cell lines U87 and T98G (Fig. 3f, g), the murine glioma cell lines GL261 (Fig. 3h), and five different neuroblastoma cell lines (Fig. 3i).

(a, b) Specific inhibition of Eg5 by dimethylenastron (DMN) leads to a dose-dependent blockade of cell proliferation in both, blood (HUVEC) and lymphatic endothelial cells (LEC). (c, d) Nearly complete inhibition is achieved in HUVECs and human brain endothelial cells (hCMEC/D3) after 72h at 1 μM (P<0.0001) in the presence of serum. (e) Eg5 inhibition blocks endothelial cell proliferation regardless the mitogen used for stimulation (FGF-2 or VEGF) in bovine aortic endothelial cells (BAE; P<0.005). (f-h) Human and murine glioma cell proliferation is inhibited with comparable efficacy. (i) Growth of five neuroblastoma cell lines is also significantly inhibited in the presence of 0.5 or 1 μM DMN.

### KIF11/Eg5 and KIF20A/Mklp2 inhibition interferes with in vitro angiogenesis in the absence of mitosis

**Figure 4:**
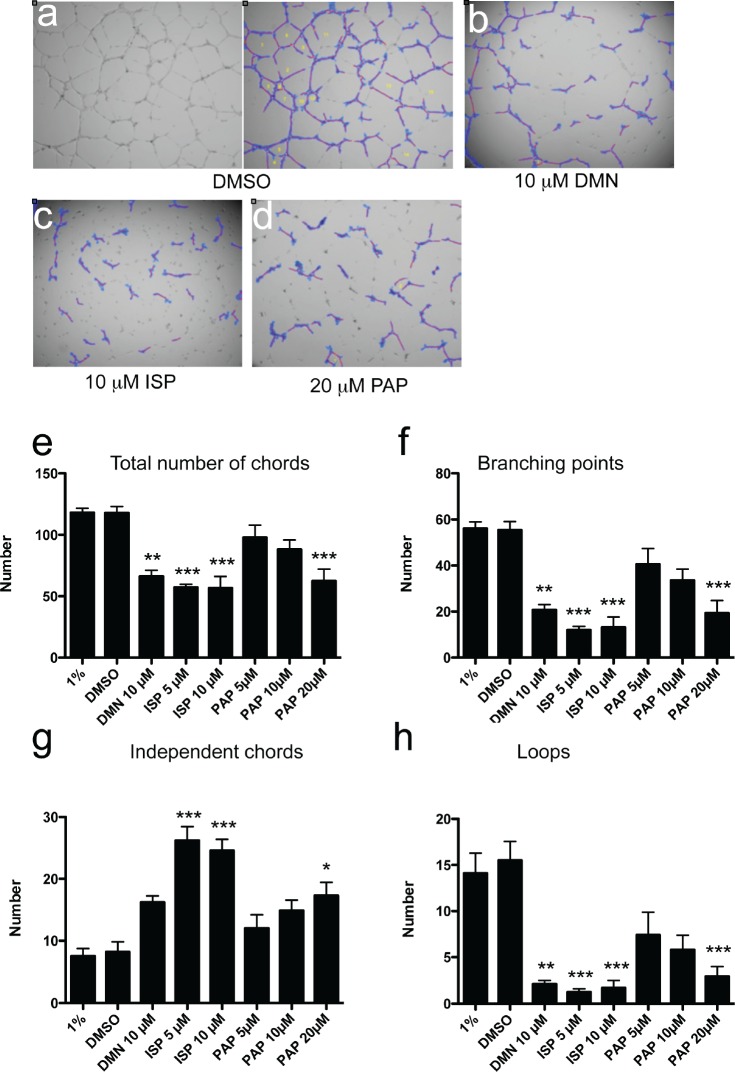
Inhibition of Eg5 or KIF20A protein function impairs in vitro angiogenesis in the absence of mitosis

Strong perturbation of in vitro angiogenesis was observed after Eg5 blockade using two specific inhibitors, dimethylenastron (DMN; Fig. 4b) and ispinesib (ISP; Fig. 4c and Video 1), whereas solvent-treated cultures were not affected (Fig. 4a and Video 2). Inhibition was observed with ispinesib at both doses (5 and 10 μM). Number of vascular chords was reduced by 2-fold, branching points by nearly 90%, independent chords increased 3-times and number of loops was reduced by 90% (Fig. 4e-h). Less potent inhibition was observed with the Eg5 inhibitor DMN (Fig. 4e-h). Chemical blockade of Mklp2/KIF20A protein using paprotrain also inhibited chord formation (Fig. 4d), albeit at higher doses (20 μM) and to a lesser extent than the KIF11/Eg5 inhibitors (Fig. 4e-h). Mitosis is a very rare event in our assay conditions (Fig. S8). These results demonstrate that kinesin inhibition affects biological processes relevant for angiogenesis, which are distinct from mitosis.

(a-d) HUVECs grown on matrigel received indicated treatments. They were photographed at 4× magnification after 14h and morphologically annotated by the WIMASIS platform (ISP = Ispinesib; DMN = dimethylenastron; PAP = paprotrain; a) left image: unprocessed raw data, right image: automated annotation by WIMASIS). (e-h) Morphological parameters characteristic of *in vitro* angiogenesis are significantly reduced by chemical inhibition of Eg5 or Mklp2 protein.

### Kinesin inhibition affects endothelial cell adhesion, spreading and migration

Vascular chord formation requires cell adhesion and migration. Eg5 function is required for proper cell adhesion of HUVECs plated on different matrix proteins. Ispinesib-treated cells (5 μM) adhered poorly and detached after washing prior to Coomassie staining (Fig. S9a). Cells appeared more rounded and phalloidin staining revealed an altered organization of the actin cytoskeleton, with an appearance of cortical actin and strong reduction of stress fibers (Fig. S9b). Quantification of the number of spreading cells revealed a significant decrease after Eg5 blockade using ispinesib (P<0.0001, Fig. S9c). Endothelial cell migration was measured over an 18h time period. Migrating HUVECs in serum and DMSO control wounds covered around 90% of the denuded area (Fig. S10a, b), whereas Ispinesib-treated cultures were covered by 36% and paprotrain cultures only by 28% (P<0.0001; Fig S10c). A comparable result was obtained in a second series of experiments after siRNA-mediated knock-down of KIF11 in HUVECs. Wound closure after 20h of migration was reduced by 31% compared to control cultures (P=0.0007; Fig. S10d).

### Vascular outgrowth in the mouse aortic ring assay is inhibited by kinesin blockade

We next tested effects of Eg5 and Mklp2 inhibition in an *ex vivo* angiogenesis model where spontaneous vascular growth occurs after incubation of aortic rings in matrigel [27]. After 8 days of treatment, vascular outgrowth around rings was significantly inhibited at ispinesib doses as low as 50nM (P<0.0001; Fig S11a, b), whereas paprotrain-mediated inhibition was effective only around 20μM (P<0.001; Fig. S11a, c).

### kif11/Eg5 is required for CAM development and physiological hematopoiesis and angiogenesis in zebrafish

**Figure 5:**
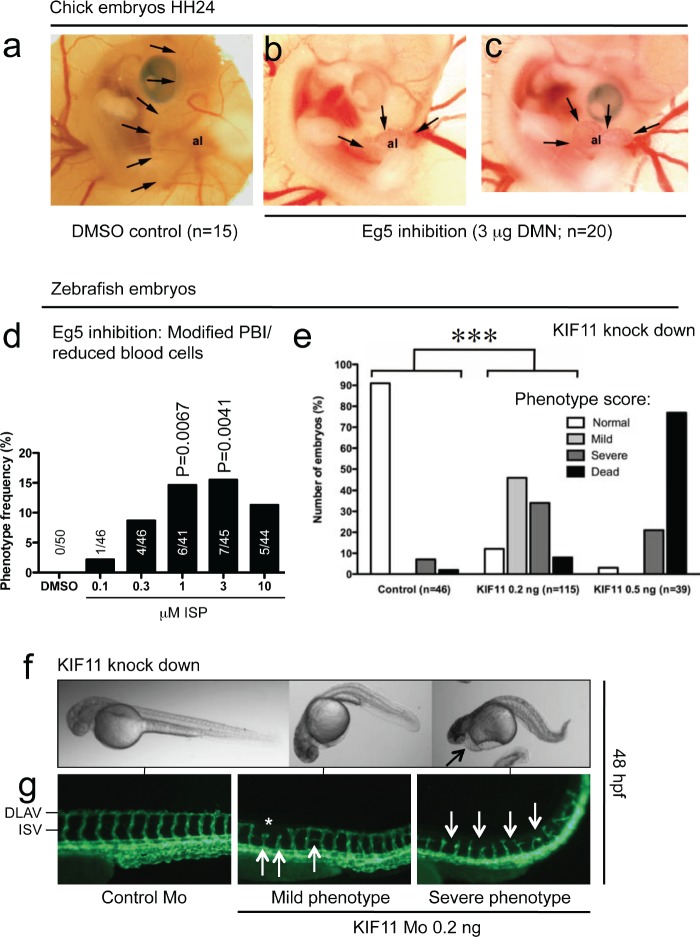
Effects of kinesin blockade in chick and zebrafish embryos (a) Control chick embryos showed normal expansion of the allantoic vesicle (al) at day 4.5 (HH24). Arrows point to the border of the vesicle. (b, c) Eg5 inhibition leads to complete arrest of CAM development; only a rudimentary tissue with a primitive vascular network develops. (d) Blockade of Eg5 function using ispinesib (ISP) from 24 to 48 hpf leads to a significant increase of embryos with modification of the posterior blood islands (PBI) or reduction of blood cells at 1 and 3μM. (e) Phenotype-score of Tg(kdrl:EGFP)^s843^ embryos injected with indicated morpholinos (Mo) at 48 hpf. At doses higher than 0.5 ng, most embryos die and show severe edema and circulation defects. (e, f) At 0.2 ng, 46% of embryos displayed a mild phenotype with a curved and shortened tail and normal circulation, 34% had severe circulation defects, including pericardial edema (arrow), and 8% of embryos were dead (70× magnification). (g) Fluorescence micrographs of control and *kif11* morphants (115× magnification). Asterisk and arrows denote random vascular defects. DLAV = dorsal longitudinal anastomotic vessel, ISV = intersomitic vessels.

DMN injection into the allantoic vesicle at Hamburger&Hamilton stage 21 (HH21) embryos completely inhibited expansion of the vesicle (HH24; Fig 5b, c). Only a rudimentary tissue mass with a primitive vascular network developed. This effect was consistent in all DMN-injected embryos, control embryos showed normal growth of the allantoic vesicle (arrows, Fig. 5a).

In normal zebrafish embryos, embryonic lethality was only observed in the ISP treatment groups, ranging from 8% at 0.1 μM, up to 18% at 1 μM. Analysis of live embryos revealed changes in the morphology of the posterior blood islands (PBI) as well as a reduction of circulating blood cells (Fig. 5d). This phenotype did not occur in control embryos, but up to 15% of embryos displayed these modifications at 1 μM (P=0.0067) and 3 μM ISP (P=0.0041), suggesting that the developing hematopoietic system is sensitive to Eg5 inhibition.

We therefore examined expression of *kif11* in 24hpf embryos; a time point where a transient wave of hematopoiesis takes place in the PBI and circulation begins [28]. A *fli1* probe visualized cellular components of the developing blood vascular system, including the PBI. The *kif11* probe labeled various cellular components of the embryo with different intensities; strong staining was evident in the PBI and PCV (posterior cardinal vein), where a partial co-localisation with the *fli1* signal was observed (Fig. S12a).

Morpholino-mediated knock down of the *kif11* gene in the transgenic Tg(kdrl:EGFP)^s843^ line had severe effects on embryonic morphology and survival (Fig. 5e). Whereas almost all control morpholino embryos developed normally, the number of dead and affected embryos increased even at a morpholino dose of 0.2 ng (P<0.005; 0.2 ng vs. controls). At 0.5 ng, effects were even more severe and 70% of embryos were dead. At the lower dose, embryos appeared curved and severe phenotypes had pericardial edema (Fig. 5f, arrow). Various morphological defects occurred in the developing vasculature, ranging from shortened inter-somitic vessel (ISV) sprouts (Fig. 5g; first two arrows), discontinuity of the dorsal longitudinal anastomotic vessel (DLAV) (asterisk, mild phenotype) and branching defects (third arrow). Embryos with severe phenotypes had complete absence of the DLAV and shortened and distorted ISV sprouts (arrows; more examples are shown in Fig. S12b).

These results – together with the high expression levels of KIF11 in hematopoietic and endothelial cells – suggest that Eg5/KIF11 might be a novel target for anti-angiogenic therapy in pathological settings.

### Interfering with KIF11/Eg5 function reduces tumor angiogenesis

**Figure 6:**
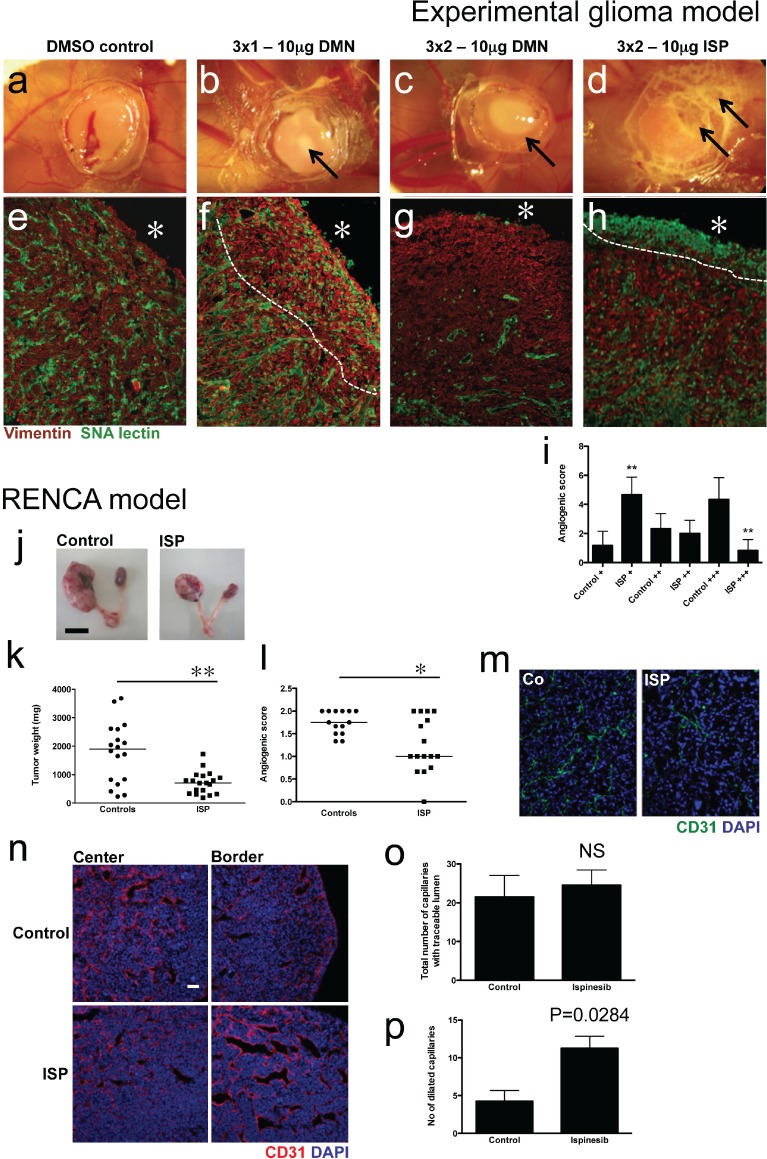
Eg5 inhibition reduces tumor angiogenesis in experimental tumor models (a-d) U87-derived gliomas on the CAM were treated with indicated doses of Eg5 inhibitors. Biomicroscopy images were taken at day 4 of tumor development. Note visible induction of necrosis (arrows in b-d) at the tumor surface after Eg5 inhibition. (e-f) Immunohistological examination of experimental glioma. A dense vascular network occurred in control tumors, whereas Eg5 inhibition leads to reduced tumor angiogenesis underneath the tumor surface (asterisk), denoted by the dashed line (f), especially at two treatments of DMN (g) or ISP (h) per day. (i) A significantly higher number of poorly vascularized tumors (+) was within the ISP-treated group, whereas few tumors treated with ISP appeared highly vascularized (+++). (j) Ispinesib also reduced tumor size (representative tumors are shown); (k) weight (P= 0.0037) and (i) vascular density (P=0.0184) in an orthotopic renal cell carcinoma model. (j). Short-term treatment (48h) with ispinesib lead to a significant increase of dilated capillaries (P=0.0284; n, p) whereas overall vascular density remained unchanged (NS; o).

Experimental gliomas grown on the chicken CAM are accessible to topical treatment with chemical tyrosine kinase inhibitors such as imatinib mesylate or PTK787 [23]. During the three-day anti-Eg5 treatment period, no measurable tumor size reduction occurred. However, biomicroscopic observation of the tumors at day 4 showed increasing whitish areas at the tumor surface (Fig. 6a-d). These areas contain necrotic cells, as confirmed by subsequent histology (Fig. 6f-h). Immunohistological analysis of the tumors showed reduced numbers of angiogenic capillaries after treatment with a single dose of DMN per day (Fig. 6f). This effect was much stronger after two DMN doses; the tumors were almost completely devoid of blood vessels, whereas tumor cells apart from the necrotic areas appeared normal (Fig. 6g). Tumor cell morphology evidenced by vimentin staining was not altered by DMN treatment, even at the highest doses. Two treatments of ispinesib caused widespread destruction of cellular components of the tumor as evidenced by non-specific staining of dead cells for *sambucus nigra* agglutinin (SNA lectin), clearly visible below the surface (Fig. 6h). A significantly higher number of ispinesib-treated tumors were found in the poorly vascularized group (P<0.005) and accordingly, only a small number of ispinesib-treated tumors were classified in the highly vascularized group (P<0.005; Fig 6i).

In a murine tumor model, RENCA cells implanted orthotopically in kidneys give rise to highly aggressive and fast growing tumors with tumor weights of up to 4 g after a 3-weeks period (median 1.9 g; Fig. 6j, k). Twice-weekly treatment with ispinesib potently reduces tumor weight (median 708 mg; P=0.0037) and tumor angiogenesis (P=0.0184; Fig. 6l, m).

When established tumors were treated for 48h with ispinesib, no growth reduction or effect on vessel density was observed (Fig. 6n, o). However, tumor vessel in treated animals appeared more dilated and had a significantly higher number of dilated vessels (Fig 6p; P=0.0284).

## DISCUSSION

It is widely accepted that the secreted growth factor VEGF-A and its transmembrane receptors, are key regulators of embryonic and pathologic angiogenesis [2]. The angiogenic switch is a key event during tumor progression [1] and there has been much hope that anti-VEGF-A therapies may inhibit tumor growth significantly. In practice, anti-VEGF strategies have their limitations, due to a limited number of therapy responders, severe side effects of the VEGF-targeting antibody Avastin and raising concerns about the high costs of such treatment [3]. However, VEGF-A is a major proangiogenic factor *in vivo*, and therefore, it is of crucial importance to identify new druggable targets, which are associated with its downstream functions.

We chose the chick embryonic day-13 chorio-allantoic membrane (CAM), since human VEGF-A specifically induces angiogenesis in this tissue [4]. A global analysis of regulated angiogenesis genes *in vivo* is more reliable than *in vitro* transcriptome analysis of angiogenic endothelial cells, since major molecular differences have been found when endothelial cells are studied separated from their normal tissue context [29, 30].

Numerous genes up-regulated by VEGF-A have not yet been studied in the context of angiogenesis such as members of the kinesin family of motor proteins. We investigated the effects of kinesin inhibition on the angiogenic process *in vitro* and *in vivo*, focusing on Eg5/KIF11 and Mklp2/KIF20A.

Our bioinformatics analysis and immunostaining results of normal human foreskin (Fig. 2) show that KIF11/Eg5 is strongly expressed in blood and lymphatic vascular ECs. Human foreskin ECs also express VEGFR-2, a marker of angiogenic capillaries [31]. Only cells from the hematopoietic lineage (normal or neoplastic), together with CD34^+^ and CD105^+^ endothelial cells also displayed high levels of KIF11 and KIF20A kinesins (Fig. S7). An observation in line with these results is the strong expression of *kif11* in the posterior blood islands of zebrafish embryos. In addition, inhibition of Eg5 function by ispinesib in this model leads to modification of this structure or reduced number of blood cells. In humans, neutropenia is a frequent side effect of anti-kinesin therapy [32], suggesting that Eg5 function is also required for generation and maintenance of immune cells.

Morpholino-mediated knock-down of *kif11* results in vascular pattern defects in the trunk vasculature (Figure 5 e-g, Fig. S12b). These results point to an important function of KIF11/Eg5 in the endothelial cell lineage.

Besides endothelial cell proliferation, other biological processes such as chord formation also require Eg5 and Mklp2. Recent insight from genetic studies further support the notion that KIF11 is required for development of normal retinal and lymphatic vessels, since KIF11 mutations cause autosomal-dominant microcephaly associated with lymphedema and/or chorioretinopathy [33].

Cell adhesion, spreading and migration that are critical for vascular chord formation are strongly perturbed by Eg5 and Mklp2 inhibition (Figs. S9, S10). We observed a modification of the cytoskeleton in cells treated with ispinesib, with an increase of cortical actin and strong reduction of stress fibers. These changes in the cytoskeleton are most likely responsible for the reduced migratory and adhesive capacity after Eg5 inhibition. Just recently, it has been shown that Eg5 also has a function in normal and malignant cell migration [14, 15]. There is evidence from the literature that DMN is highly specific for Eg5 kinesin, it binds to a region of the protein composed of loop 5, helix α2 and α3, which is not conserved in other kinesins [34, 35]. ISP, the second inhibitor we used ISP has been shown to be more than 70.000-times more selective for Eg5 than for other kinesins [36]. Further, siRNA-mediated knockdown of the KIF11 gene reduced cell migration comparable to chemical inhibition.

Interestingly, all five kinesins induced by VEGF-A are also present in the highly significant 67-gene signature termed CINSARC (complexity index in sarcomas), which is associated with poor clinical outcome in several cancer types [37]. It is tempting to speculate that VEGF-A-induced kinesins contributes to bad prognosis in human cancers or reflects the state of an activated tumor endothelium. Significant co-expression of these five kinesins has also been evidenced by bioinformatic analysis in various malignant tumors, especially in brain tumors (Fig. S5). In hepatocellular carcinoma (HCC), increasing kinesin (co-) expression is furthermore associated with the vascular status of the tumors (Fig. S6). Another group has just recently confirmed overexpression of KIF20A in HCC [38].

As expected, Eg5 inhibition stops glioblastoma cell growth [39]. Eg5 inhibitors not only block proliferation of adult tumor cell lines, but also those derived from childhood tumors. In the progressed stage 4 neuroblastoma, the 5-year survival rate is only 20 – 30% [40] and high vascular density is characteristic for the progressed neuroblastomas [41]. Further studies should investigate the efficacy of anti-kinesin treatment in this kind of malignancy.

In the light of our *in vitro* results on Eg5/KIF11 inhibition of endothelial cells, it can be expected that Eg5 inhibition might have a dual effect as it affects both malignant cells and tumor vasculature. Inhibition of tumor angiogenesis in the short-term experimental glioma model [23] was the predominant effect observed, suggesting that the endothelium constitutes an important target for this inhibitor.

Oncomine expression data show that KIF11 transcripts are not overexpressed in kidney cancer, however, we have found Eg5 protein expression in capillaries of RCC and just recently, Eg5 immunoreactivity has been evidenced in a large study using 164 patient RCC tumors, with higher expression predicting poor disease outcome [42]. Eg5 inhibition significantly reduces experimental kidney cancer growth and angiogenesis. Short-term anti-Eg5 treatment did not alter tumor vessel density or led to regression of vessels, but changed vascular morphology. Tumor capillaries were significantly more dilated in the treatment group, suggesting a vascular remodelisation effect of ispinesib. Even though classical anti-VEGF agents generally causes vascular “normalization” including reduction of vessel diameter [43], dilatation of tumor capillaries after different anti-angiogenic treatments has recently described in animal models as well in human tumors [44, 45]. Different treatment durations and drug combinations may account for these differences; however, our results show that the tumor vasculature is affected by Eg5 inhibition.

This may open up new therapeutic perspectives such as targeting kinesin inhibitors to the tumor endothelium and stroma [46] to enhance the therapeutic efficacy.

Furthermore, combination of small-molecule Eg5 inhibitors with anti-angiogenic agents may reduce side effects or increase the number of responders to anti-angiogenesis treatment. Eg5 blockade not only inhibits VEGF-A-induced cell proliferation, but also FGF-2 stimulated proliferation and thus has a more broad-range effect than specific VEGF inhibitors (Fig. 3e). It is therefore tempting to speculate that Eg5 inhibition might render endothelial cells refractory to growth stimuli secreted by tumor cells, thereby maintaining a potent anti-angiogenic environment.

Even though single anti-angiogenic treatment works well in animal models, combination with standard chemotherapy is needed for most tumor types to obtain results in patients. A special emphasis should be given to the fact that cells from the hematopoietic lineage overexpress Eg5 (and Mklp2). Patients suffering from these highly proliferative malignancies might benefit from kinesin inhibition due to its dual anti-proliferative and anti-angiogenic effect. Since a frequent side effect of Eg5 inhibitors is neutropenia, efforts should be made to develop specific cell and tumor targeting kinesin-inhibition strategies to enhance their efficacy.

Evidence is emerging that over expression of KIF4A and KIF18A may play roles in tumor progression [47, 48]. Furthermore, knock-down of the KIF23 gene has been shown to abrogate glioma cell proliferation and tumor growth in vivo [49]. This opens the perspective that other kinesins may serve as druggable targets for anti-cancer therapy.

Taken together, our results provide evidence that kinesins, which lay downstream of angiogenic growth factors signaling, mediate essential processes important for physiological and pathological vascular growth and may constitute a potential therapeutic target for anti-vascular tumor therapy.

## MATERIAL AND METHODS

Detailed protocols are in the supplemental data section.

### Identification of genes regulated by VEGF-A on the CAM

Chick embryos were handled as described [18]. RNA was extracted from three CAMs treated each with 3μg of recombinant human VEGF-A and compared individually to pooled equivalent quantities of RNA from three normal CAMs. Affymetrix chicken microarrays were processed as published [19]. Bioinformatic analyses of kinesin expression are detailed in the supplemental data section.

### Semi-quantitative PCR

To verify microarray data, semi-quantitative PCR was performed on the same material used for microarray hybridization using efficient primers against VEGFR1, VEGFR2, KIF4A, KIF11, KIF15, KIF20A and KIF23, normalized to HNRPH1, which shows no changes during normal CAM development [19] and which is not induced by VEGF (Supplemental Table S1, last line).

### Chemical inhibitors

Indirubin-3'-monoxime (IRO, CDK/GSK-3β inhibitor) [20] was purchased from Sigma, dimethylenastron (DMN, Eg5 inhibitor) was from Sigma or Chemstep Molexplorer (Martillac, France), ispinesib mesylate (ISP, Eg5 inhibitor) was from Selleck Chemicals (Houston, Tx, USA). (Z)-2-(1H-indol-3-yl)-3-(pyridin-3-yl)acrylonitrile (paprotrain = PAssenger PROteins TRAnsport INhibitor, MKLP2 inhibitor) [21] was synthesized by Chemstep Molexplorer.

### Cell culture and proliferation assays

Vascular endothelial cells (HUVECs, BAEs, hCMEC/D3 and tumor cell lines (U87, T98G, GL261, CHP134, GIMEN, Kelly, IMR5 and SHEP) were cultured using routine cell culture conditions.

### Endothelial cell adhesion, spreading, migration and tubulogenesis assays

These assays were performed with HUVECs using standard protocols (see supplemental methods).

### Mouse aortic ring assays

Vascular outgrowth in this assay was evaluated using freshly isolated mouse aortic ring fragments (for details see supplemental methods).

### Chemical inhibition of Eg5 in chick embryos

Chick embryos were injected with 3 μl of dimethylenastron (1 μg/μl) at Hamburger & Hamilton (HH) stage 21 (3.5 days) into the allantoic vesicle (n=20 embryos). Control embryos (n=15) received equal volume of solvent (DMSO). Embryos were examined under a biomicroscope the following day at stage HH24.

### Zebrafish experiments

All zebrafish experiments were carried out using standard procedures (described in supplemental material and methods). In brief, Tg(kdrl:EGFP)^s843^ embryos [22] were microinjected at the 1-2 cell stage with 0.2-1 ng *kif11* morpholino (MO) (CTGGTACTTGTGATGATGCCATGTT; Gene Tools, USA) or standard control MO (CCTCTTACCTCAGTTACAATTTATA; Gene Tools, USA). Higher *kif11* MO doses were lethal and did not allow further analysis. At 48 hours post-fertilization (hpf), embryos were phenotype-scored and photographed. For chemical Eg5 inhibition, normal zebrafish embryos were treated with indicated doses of ispinesib at 24 hpf and phenotype-scored at 48 hpf.

### Experimental tumor models

U87 glioma cells were implanted on the CAM as described [23] and treated once or twice daily with DMN or ISP, at indicated doses. Control tumors received solvent (DMSO). Tumors were photographed *in vivo* and blind-scored by seven investigators familiar with the model for degree of vascularization [24], and processed for histology. The orthotopic renal carcinoma model is detailed in the supplemental data section.

### Histology, immunohistology and fluorescence in situ hybridization

Unfixed human foreskin from healthy donors was used for Eg5 (polyclonal rabbit anti-human, Abcam ab37009) co-localization studies with lymphatic (mouse-anti-human podoplanin, ReliaTech, Germany) and vascular endothelium (mouse anti human CD31; BD clone WM59). For analysis of experimental glioma, anti-vimentin (clone V9, Neomarkers) was used. Chick blood vessels were stained with *sambucus nigra* lectin (SNA, Vector Labs), and nuclei counterstained with DAPI. For *in situ* hybridization, fluorescent zebrafish probes *kif11* and *fli1* were used on whole embryos. Human tumor sections were analyzed for Eg5 expression by standard immunohistochemistry (www.proteinatlas.org) and for KIF20A expression using QuantiViewRNA hybridization (Panomics-Affymetrix), details are in the supplemental methods section.

## Supplementary Figures and Tables










