# Supplementary material for: Impaired angiogenesis and tumor development by inhibition of the mitotic kinesin Eg5
Source: Oncotarget. 2013 Oct 26;4(12):2302–16. doi: 10.18632/oncotarget.1490 (PMC3926828; doi:10.18632/oncotarget.1490)
Supplement: Supplementary file 1 [file oncotarget-04-2302-s001.pdf]

Table S1

Complete list of 317 CAM genes induced by VEGF

(provided as Microsoft Excel file)

The table contains the list of 317 probe sets detecting significantly up regulated transcripts in at least two out of three biological replicates. The five kinesins motor protein encoding genes are marked in red (KIF4A, KIF11, KIF15, KIF20A and KIF23).
I = Increased.

Figure S1

Expression of Eg5 protein in endothelial cells is stimulated by VEGF-A *in vitro* and inhibited by Avastin

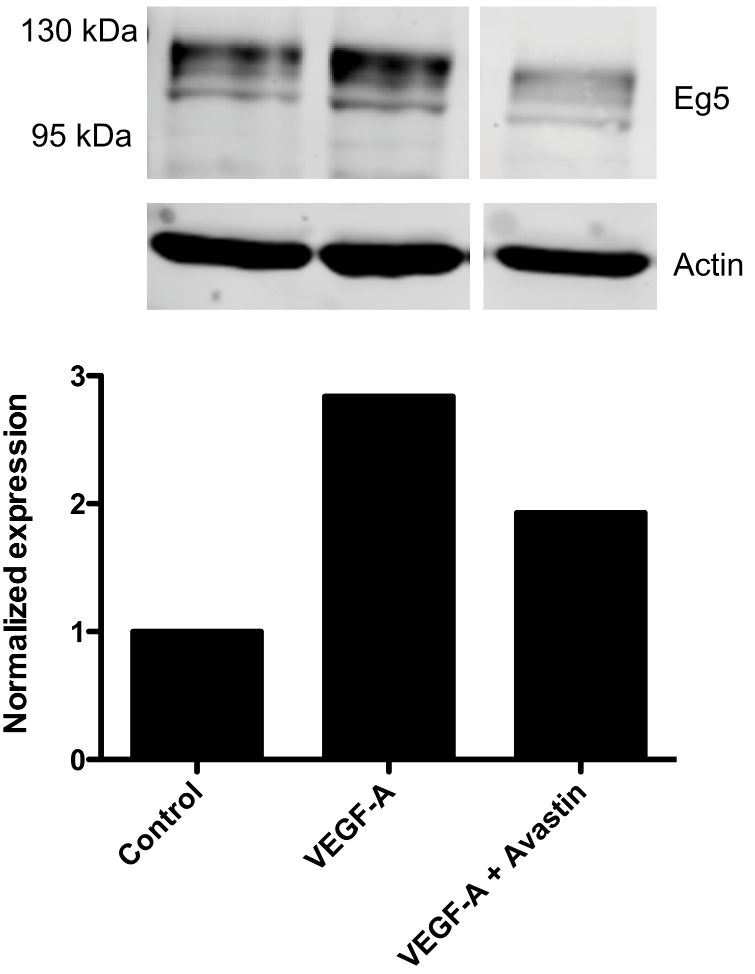

HUVECs were treated with 10 ng/ml recombinant human VEGF-A for 24 h and Eg5 protein expression is analyzed by Western Blot. Note that co-treatment with VEGF-A blocking antibody Avastin abolishes Eg5 expression.

Table S2

Biological themes significantly enriched in genes up regulated by VEGF

Annotation Cluster 1	Enrichment Score: 11.6											
Category	Term	Count	%	PValue	Genes	List Total	Pop Hits	Pop Total	Fold Enrichment	Bonferroni	Benjamini	FDR
SP_PIR_KEYWORDS	mitosis	22	10.68	5.37E-16	KIF23, KIF11, CDC2, NUF2, NUSAP1, NDC80, CENPE, CDC20, SMC2, SMC4, NCAPD2, FAM83D, NCAPH, CCNB2, PLK1, NCAPG, NCAPG2, BUB1, BUB1B, CCNA2, HELLS, CDCA3	206	183	19235	11.22526	1.68E-13	1.68E-13	7.44E-13
GOTERM_BP_FAT	GO:0048285~organelle fission	25	12.136	1.53E-15	KIF23, FAM83D, NCAPH, NCAPG2, NCAPG, BUB1, CCNA2, HELLS, CDCA3, KIF11, OPA1, CDC2, KIF15, NUF2, TPX2, NUSAP1, CENPE, NDC80, CDC20, SMC2, NCAPD2, SMC4, CCNB2, PLK1, BUB1B	174	229	13528	8.487678	2.64E-12	2.64E-12	2.62E-12
GOTERM_BP_FAT	GO:0000280~nuclear division	24	11.65	6.74E-15	KIF23, KIF11, CDC2, KIF15, TPX2, NUF2, NUSAP1, NDC80, CENPE, CDC20, SMC2, SMC4, NCAPD2, FAM83D, NCAPH, CCNB2, PLK1, NCAPG, NCAPG2, BUB1, BUB1B, CCNA2, HELLS, CDCA3	174	220	13528	8.481505	1.15E-11	5.74E-12	1.14E-11
GOTERM_BP_FAT	GO:0007067~mitosis	24	11.65	6.74E-15	KIF23, KIF11, CDC2, KIF15, TPX2, NUF2, NUSAP1, NDC80, CENPE, CDC20, SMC2, SMC4, NCAPD2, FAM83D, NCAPH, CCNB2, PLK1, NCAPG, NCAPG2, BUB1, BUB1B, CCNA2, HELLS, CDCA3	174	220	13528	8.481505	1.15E-11	5.74E-12	1.14E-11
GOTERM_BP_FAT	GO:0000087~M phase of mitotic cell cycle	24	11.65	9.98E-15	KIF23, KIF11, CDC2, KIF15, TPX2, NUF2, NUSAP1, NDC80, CENPE, CDC20, SMC2, SMC4, NCAPD2, FAM83D, NCAPH, CCNB2, PLK1, NCAPG, NCAPG2, BUB1, BUB1B, CCNA2, HELLS, CDCA3	174	224	13528	8.330049	1.69E-11	5.65E-12	1.69E-11
SP_PIR_KEYWORDS	cell cycle	30	14.563	1.36E-14	KIF23, E2F1, FAM83D, NCAPH, CDC45L, NCAPG2, NCAPG, BUB1, CCNA2, HELLS, CDCA3, CKAP2, RBBP4, KIF11, CDC2, NUF2, NUSAP1, CENPE, NDC80, CDC20, MCM2, SMC2, SMC4, GAK, MCM6, NCAPD2, CCNB2, RGS2, PLK1, BUB1B	206	461	19235	6.076385	4.10E-12	2.05E-12	1.81E-11
GOTERM_BP_FAT	GO:0000279~M phase	27	13.107	8.52E-14	KIF23, FAM83D, NCAPH, NCAPG2, NCAPG, BUB1, CCNA2, HELLS, CDCA3, MSH6, KIF11, CDC2, KIF15, NUF2, TPX2, NUSAP1, CENPE, NDC80, CDC20, SMC2, SMC4, RAD51, NCAPD2, CCNB2, PLK1, TOP3A, BUB1B	174	329	13528	6.380463	1.44E-10	3.61E-11	1.44E-10
SP_PIR_KEYWORDS	cell division	23	11.165	9.44E-14	KIF23, KIF11, CDC2, NUF2, NUSAP1, NDC80, CENPE, CDC20, SMC2, MCM5, SMC4, NCAPD2, FAM83D, NCAPH, CCNB2, PLK1, NCAPG, NCAPG2, BUB1, BUB1B, CCNA2, HELLS, CDCA3	206	264	19235	8.134837	2.86E-11	7.15E-12	1.26E-10
GOTERM_BP_FAT	GO:0007049~cell cycle	39	18.932	4.48E-13	KIF23, E2F1, FAM83D, NCAPH, CDC45L, NCAPG2, NCAPG, PSMD1, BUB1, CCNA2, HELLS, CDCA3, CKAP2, GPS1, MSH6, RBBP4, KIF11, UPF1, CDC2, KIF15, TPX2, NUF2, NUSAP1, CDC20, CENPE, NDC80, MCM2, SMC2, SMC4, GAK, NCAPD2, RAD51, MCM6, CCNB2, RGS2, PLK1, TOP3A, BUB1B, BARD1	174	776	13528	3.907394	7.60E-10	1.52E-10	7.56E-10
GOTERM_BP_FAT	GO:0022403~cell cycle phase	28	13.592	2.69E-12	KIF23, E2F1, FAM83D, NCAPH, NCAPG2, NCAPG, BUB1, CCNA2, HELLS, CDCA3, MSH6, KIF11, CDC2, KIF15, NUF2, TPX2, NUSAP1, CENPE, NDC80, CDC20, SMC2, SMC4, RAD51, NCAPD2, CCNB2, PLK1, TOP3A, BUB1B	174	414	13528	5.25826	4.56E-09	7.60E-10	4.53E-09
GOTERM_BP_FAT	GO:0000278~mitotic cell cycle	26	12.621	8.72E-12	KIF23, E2F1, FAM83D, NCAPH, NCAPG2, NCAPG, PSMD1, BUB1, CCNA2, HELLS, CDCA3, KIF11, CDC2, KIF15, NUF2, TPX2, NUSAP1, CENPE, NDC80, CDC20, SMC2, NCAPD2, SMC4, CCNB2, PLK1, BUB1B	174	370	13528	5.463312	1.48E-08	2.11E-09	1.47E-08
GOTERM_BP_FAT	GO:0051301~cell division	23	11.165	2.54E-11	KIF23, KIF11, CDC2, NUF2, NUSAP1, NDC80, CENPE, CDC20, SMC2, MCM5, SMC4, NCAPD2, FAM83D, NCAPH, CCNB2, PLK1, NCAPG, NCAPG2, BUB1, BUB1B, CCNA2, HELLS, CDCA3	174	295	13528	6.06164	4.31E-08	5.39E-09	4.29E-08
GOTERM_BP_FAT	GO:0022402~cell cycle process	30	14.563	1.33E-10	KIF23, E2F1, FAM83D, NCAPH, NCAPG2, NCAPG, PSMD1, BUB1, CCNA2, HELLS, CDCA3, MSH6, KIF11, CDC2, KIF15, TPX2, NUF2, NUSAP1, CENPE, NDC80, CDC20, SMC2, SMC4, RAD51, NCAPD2, CCNB2, PLK1, TOP3A, BUB1B, BARD1	174	565	13528	4.128166	2.26E-07	2.51E-08	2.24E-07
GOTERM_CC_FAT	GO:0000793~condensed chromosome	13	6.3107	6.76E-08	NUF2, NDC80, CENPE, SMC2, RAD51, NCAPD2, SMC4, NCAPH, BAZ1B, NCAPG, CENPC1, BUB1, BUB1B	160	129	12782	8.050678	1.99E-05	9.93E-06	9.00E-05
GOTERM_CC_FAT	GO:0005819~spindle	13	6.3107	2.86E-07	CKAP2, KIF23, KIF11, CDC2, KIF15, TPX2, NUSAP1, CDC20, CENPE, FAM83D, PLK1, BUB1, BUB1B	160	147	12782	7.064881	8.40E-05	2.80E-05	3.80E-04
**Annotation Cluster 2**	**Enrichment Score: 8.2**											
Category	Term	Count	%	PValue	Genes	List Total	Pop Hits	Pop Total	Fold Enrichment	Bonferroni	Benjamini	FDR
GOTERM_BP_FAT	GO:0000279~M phase	27	13.107	8.52E-14	KIF23, FAM83D, NCAPH, NCAPG2, NCAPG, BUB1, CCNA2, HELLS, CDCA3, MSH6, KIF11, CDC2, KIF15, NUF2, TPX2, NUSAP1, CENPE, NDC80, CDC20, SMC2, SMC4, RAD51, NCAPD2, CCNB2, PLK1, TOP3A, BUB1B	174	329	13528	6.380463	1.44E-10	3.61E-11	1.44E-10
GOTERM_BP_FAT	GO:0007049~cell cycle	39	18.932	4.48E-13	KIF23, E2F1, FAM83D, NCAPH, CDC45L, NCAPG2, NCAPG, PSMD1, BUB1, CCNA2, HELLS, CDCA3, CKAP2, GPS1, MSH6, RBBP4, KIF11, UPF1, CDC2, KIF15, TPX2, NUF2, NUSAP1, CDC20, CENPE, NDC80, MCM2, SMC2, SMC4, GAK, NCAPD2, RAD51, MCM6, CCNB2, RGS2, PLK1, TOP3A, BUB1B, BARD1	174	776	13528	3.907394	7.60E-10	1.52E-10	7.56E-10
GOTERM_CC_FAT	GO:0005694~chromosome	23	11.165	5.67E-08	MSH6, RBBP4, CENPL, UPF1, NUF2, NDC80, CENPE, MCM2, SMC2, SMC4, RAD51, NCAPD2, NCAPH, BAZ1B, NCAPG, CENPC1, HNRNPD, TOP3A, BUB1, BUB1B, BCL6, TOP2A, HELLS	160	460	12782	3.994375	1.67E-05	1.67E-05	7.56E-05

(table continues on next page)

Table S2

Biological themes significantly enriched in genes up regulated by VEGF

Annotation Cluster 3	Enrichment Score: 6.7											
Category	Term	Count	%	PValue	Genes	List Total	Pop Hits	Pop Total	Fold Enrichment	Bonferroni	Benjamini	FDR
UP_SEQ_FEATURE	nucleotide phosphate-binding region:ATP	35	16.99	7.84E-10	KIF23, FES, MYO9A, N4BP2, LONP1, TEK, BUB1, TOP2A, NSF, HELLS, SYK, MSH6, KIF11, FLT1, UPF1, MYO1C, LYN, CDC2, KIF15, CENPE, MCM2, SMC2, MCM4, MCM5, SMC4, ABCG2, RAD51, MCM6, KDR, PLK1, HIPK2, BMP2K, BUB1B, JAK2, KIF20A	206	962	19113	3.375628	6.53E-07	6.53E-07	1.21E-06
SP_PIR_KEYWORDS	atp-binding	40	19.417	5.88E-09	KIF23, FES, MYO9A, N4BP2, LONP1, TEK, BUB1, TOP2A, NSF, HELLS, SYK, MSH6, KIF11, FLT1, UPF1, MYO1C, LYN, CDC2, KIF15, CENPE, RBKS, MCM2, MCM4, SMC2, MCM5, SMC4 [illegible]	206	1326	19235	2.816705	1.78E-06	3.56E-07	7.87E-06
SP_PIR_KEYWORDS	nucleotide-binding	45	21.845	1.91E-08	KIF23, GNAI2, FES, MYO9A, N4BP2, LONP1, TEK, LSG1, BUB1, RAPGEF3, TOP2A, NSF, HELLS, SYK, MSH6, KIF11, FLT1, UPF1, OPA1, MYO1C, LYN, CDC2, KIF15, RBKS, CENPE, MCM2, MCM4, SMC2, MCM5, SMC4, GAK, KDR, ABCG2, RAD51, MCM6, BAZ1B, PLK1, HIPK2, RAB22A, RRM1, BMP2K, TOP3A, BUB1B, JAK2, KIF20A	206	1686	19235	2.492183	5.79E-06	9.65E-07	2.56E-05
GOTERM_MF_FAT	GO:0030554~adenyl nucleotide binding	44	21.359	1.59E-07	KIF23, STEAP4, FES, MYO9A, N4BP2, LONP1, TEK, BUB1, RAPGEF3, TOP2A, NSF, HELLS, DUS3L, SYK, MSH6, KIF11, FLT1, UPF1, MYO1C, LYN, CDC2, KIF15, TPX2, CENPE, RBKS, MCM2, MCM4, SMC2, MCM5, SMC4, GAK, KDR, ABCG2, RAD51, MCM6, BAZ1B, PLK1, HIPK2, RRM1, BMP2K, TOP3A, BUB1B, JAK2, KIF20A	158	1577	12983	2.292656	5.93E-05	5.93E-05	2.20E-04
GOTERM_MF_FAT	GO:0001883~purine nucleoside binding	44	21.359	2.44E-07	KIF23, STEAP4, FES, MYO9A, N4BP2, LONP1, TEK, BUB1, RAPGEF3, TOP2A, NSF, HELLS, DUS3L, SYK, MSH6, KIF11, FLT1, UPF1, MYO1C, LYN, CDC2, KIF15, TPX2, CENPE, RBKS, MCM2, MCM4, SMC2, MCM5, SMC4, GAK, KDR, ABCG2, RAD51, MCM6, BAZ1B, PLK1, HIPK2, RRM1, BMP2K, TOP3A, BUB1B, JAK2, KIF20A	158	1601	12983	2.258288	9.09E-05	4.54E-05	3.36E-04
GOTERM_MF_FAT	GO:0001882~nucleoside binding	44	21.359	2.95E-07	KIF23, STEAP4, FES, MYO9A, N4BP2, LONP1, TEK, BUB1, RAPGEF3, TOP2A, NSF, HELLS, DUS3L, SYK, MSH6, KIF11, FLT1, UPF1, MYO1C, LYN, CDC2, KIF15, TPX2, CENPE, RBKS, MCM2, MCM4, SMC2, MCM5, SMC4, GAK, KDR, ABCG2, RAD51, MCM6, BAZ1B, PLK1, HIPK2, RRM1, BMP2K, TOP3A, BUB1B, JAK2, KIF20A	158	1612	12983	2.242878	1.10E-04	3.67E-05	4.08E-04
GOTERM_MF_FAT	GO:0032559~adenyl ribonucleotide binding	42	20.388	3.03E-07	KIF23, FES, MYO9A, N4BP2, LONP1, TEK, BUB1, RAPGEF3, TOP2A, NSF, HELLS, SYK, MSH6, KIF11, FLT1, UPF1, MYO1C, LYN, CDC2, KIF15, TPX2, CENPE, RBKS, MCM2, MCM4, SMC2, MCM5, SMC4, GAK, KDR, ABCG2, RAD51, MCM6, BAZ1B, PLK1, HIPK2, RRM1, BMP2K, TOP3A, BUB1B, JAK2, KIF20A	158	1497	12983	2.305396	1.13E-04	2.83E-05	4.19E-04
GOTERM_MF_FAT	GO:0005524~ATP binding	41	19.903	5.95E-07	KIF23, FES, MYO9A, N4BP2, LONP1, TEK, BUB1, TOP2A, NSF, HELLS, SYK, MSH6, KIF11, FLT1, UPF1, MYO1C, LYN, CDC2, KIF15, TPX2, CENPE, RBKS, MCM2, MCM4, SMC2, MCM5, SMC4, GAK, KDR, ABCG2, RAD51, MCM6, BAZ1B, PLK1, HIPK2, RRM1, BMP2K, TOP3A, BUB1B, JAK2, KIF20A	158	1477	12983	2.280979	2.22E-04	4.44E-05	8.22E-04
**Annotation Cluster 4**	**Enrichment Score: 5.5**											
Category	Term	Count	%	PValue	Genes	List Total	Pop Hits	Pop Total	Fold Enrichment	Bonferroni	Benjamini	FDR
GOTERM_BP_FAT	GO:0001568~blood vessel development	17	8.2524	9.84E-08	CCM2, FLT1, LMO2, COL3A1, ELK3, PROX1, GJA5, CDH5, KDR, CDH13, MEOX2, NARG1, ID1, FOXC2, SOX18, COL1A1, SOX17	174	245	13528	5.394699	1.67E-04	1.52E-05	1.66E-04
GOTERM_BP_FAT	GO:0001944~vasculature development	17	8.2524	1.38E-07	CCM2, FLT1, LMO2, COL3A1, ELK3, PROX1, GJA5, CDH5, KDR, CDH13, MEOX2, NARG1, ID1, FOXC2, SOX18, COL1A1, SOX17	174	251	13528	5.265742	2.34E-04	1.95E-05	2.32E-04
**Annotation Cluster 5**	**Enrichment Score: 5.4**											
Category	Term	Count	%	PValue	Genes	List Total	Pop Hits	Pop Total	Fold Enrichment	Bonferroni	Benjamini	FDR
GOTERM_BP_FAT	GO:0030261~chromosome condensation	8	3.8835	1.96E-08	NCAPH, NCAPG2, NCAPG, NUSAP1, TOP2A, SMC2, NCAPD2, SMC4	174	25	13528	24.87908	3.33E-05	3.33E-06	3.31E-05
GOTERM_CC_FAT	GO:0005694~chromosome	23	11.165	5.67E-08	MSH6, RBBP4, CENPL, UPF1, NUF2, NDC80, CENPE, MCM2, SMC2, SMC4, RAD51, NCAPD2, NCAPH, BAZ1B, NCAPG, CENPC1, HNRNPD, TOP3A, BUB1, BUB1B, BCL6, TOP2A	160	460	12782	3.994375	1.67E-05	1.67E-05	7.56E-05
GOTERM_CC_FAT	GO:0000793~condensed chromosome	13	6.3107	6.76E-08	NUF2, NDC80, CENPE, SMC2, RAD51, NCAPD2, SMC4, NCAPH, BAZ1B, NCAPG, CENPC1, BUB1, BUB1B	160	129	12782	8.050678	1.99E-05	9.93E-06	9.00E-05
SP_PIR_KEYWORDS	dna condensation	6	2.9126	9.76E-08	NCAPH, NCAPG2, NCAPG, SMC2, NCAPD2, SMC4	206	12	19235	46.68689	2.96E-05	4.22E-06	1.31E-04
GOTERM_BP_FAT	GO:0000070~mitotic sister chromatid segregation	8	3.8835	3.02E-07	NCAPH, NCAPG, NUSAP1, CENPE, NDC80, SMC2, NCAPD2, SMC4	174	36	13528	17.27714	5.13E-04	3.95E-05	5.10E-04
GOTERM_CC_FAT	GO:0000796~condensin complex	5	2.4272	3.39E-07	NCAPH, NCAPG, SMC2, NCAPD2, SMC4	160	6	12782	66.57292	9.97E-05	2.49E-05	4.52E-04
GOTERM_BP_FAT	GO:0000819~sister chromatid segregation	8	3.8835	3.69E-07	NCAPH, NCAPG, NUSAP1, CENPE, NDC80, SMC2, NCAPD2, SMC4	174	37	13528	16.81019	6.26E-04	4.47E-05	6.22E-04
GOTERM_BP_FAT	GO:0007076~mitotic chromosome condensation	6	2.9126	3.82E-07	NCAPH, NCAPG, NUSAP1, SMC2, NCAPD2, SMC4	174	13	13528	35.88329	6.48E-04	4.32E-05	6.45E-04

Out of the 317 probe sets, which detected up regulated transcripts in VEGF-treated CAMs, 206 unique orthologs were submitted to gene ontology analysis. Note that significantly enriched GO-terms identify cell proliferation and blood vessel development as main biological processes (>5-fold enrichment, FDR<0.001).

Table S3

In silico prediction of endothelial versus non-endothelial enrichment of human kinesin family members

Gene symbol	Gene name	No. EC-EST	No. Non EC-EST	Enrichment EC-EST/Non EC-EST	FDR q-value	
KIF11	kinesin family member 11	2	0	-	0.10	Endothelial
KIF13B	kinesin family member 13B	3	2	6.59	0.26	
KIF18A	kinesin family member 18A	2	2	4.39	0.44	
KIF9	kinesin family member 9	1	1	4.39	0.57	
KIF15	kinesin family member 15	1	1	4.39	0.57	
KIF20A	kinesin family member 20A	5	6	3.66	0.26	
KIF2C	kinesin family member 2C	3	6	2.20	0.57	
KIF13A	kinesin family member 13A	1	2	2.20	0.62	
KIF1B	kinesin family member 1B	2	5	1.76	0.62	
KIF5B	kinesin family member 5B	3	8	1.65	0.62	
KIF3A	kinesin family member 3A	1	3	1.46	0.81	
KIF1C	kinesin family member 1C	1	4	1.10	0.94	
KIF22	kinesin family member 22	2	14	1.59	0.62	Non-Endothelial
KIF12	kinesin family member 12	0	3	-	0.53	
KIF17	kinesin family member 17	0	1	-	0.62	
KIF1A	kinesin family member 1A	0	3	-	0.53	
KIF21A	kinesin family member 21A	0	2	-	0.57	
KIF21B	kinesin family member 21B	0	4	-	0.49	
KIF23	kinesin family member 23	0	1	-	0.62	
KIF24	kinesin family member 24	0	1	-	0.62	
KIF27	kinesin family member 27	0	1	-	0.62	
KIF3B	kinesin family member 3B	0	7	-	0.33	
KIF3C	kinesin family member 3C	0	4	-	0.49	
KIF4A	kinesin family member 4A	0	3	-	0.53	
KIF5C	kinesin family member 5C	0	9	-	0.29	
KIFC1	kinesin family member C1	0	2	-	0.57	
KIFC2	kinesin family member C2	0	2	-	0.57	
KIFC3	kinesin family member C3	0	8	-	0.29	
				(no restriction for q-value)		

Genes encoding for kinesins were extracted from the Affymetrix chicken GeneChip by their gene symbol (KIFxx), subjected to ortholog screen and *in silico* assignment of endothelial vs. non-endothelial enrichment. Out of 36 unique kinesin genes, 12 showed preferential endothelial expression, whereas 16 were enriched in non-endothelial cells.

Figure S2

Expression and co-expression analysis of five VEGF-induced kinesins in various human tumors

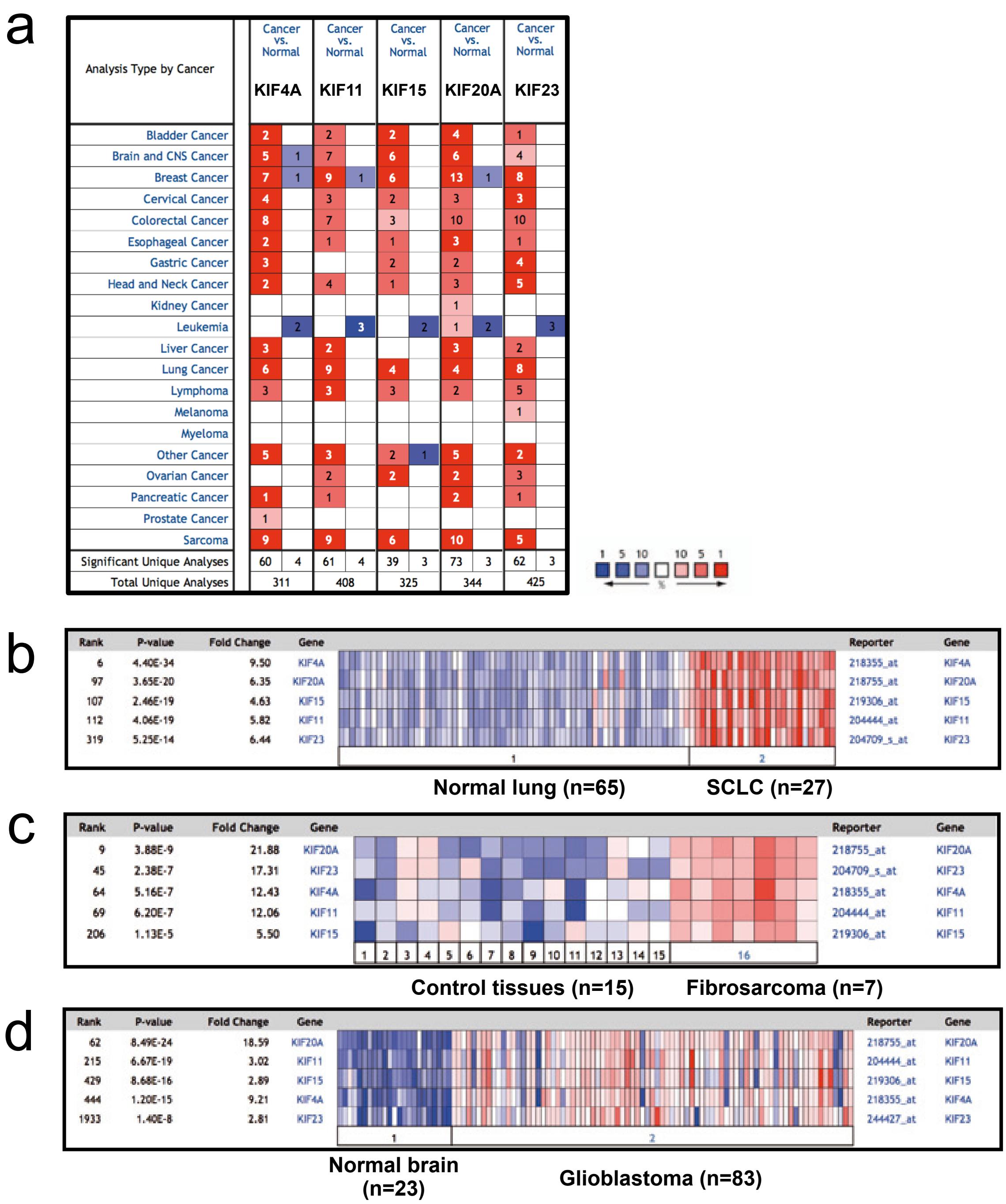

(a) Oncomine database was queried for expression of indicated kinesins in human malignancies compared to normal tissue. The numbers in the cases indicated in how many different studies significant over expression was found. Intensity of the red and blue colour indicates rank-order of the gene by over expression and under expression in cancer versus normal tissue (eg. top 1%, 5% and 10%). Co-expression of five VEGF-induced kinesins was observed in three different cancer types. In all cases, kinesins were significantly over expressed in cancer tissues. (b) SCLC = squamous cell lung carcinoma; Hou_Lung study. (c) Fibrosarcoma; Detwiller_sarcoma study. (d) Glioblastoma; Sun_Brain study.

Figure S3

Heterogeneous expression of KIF11 and KIF20A in glioblastoma

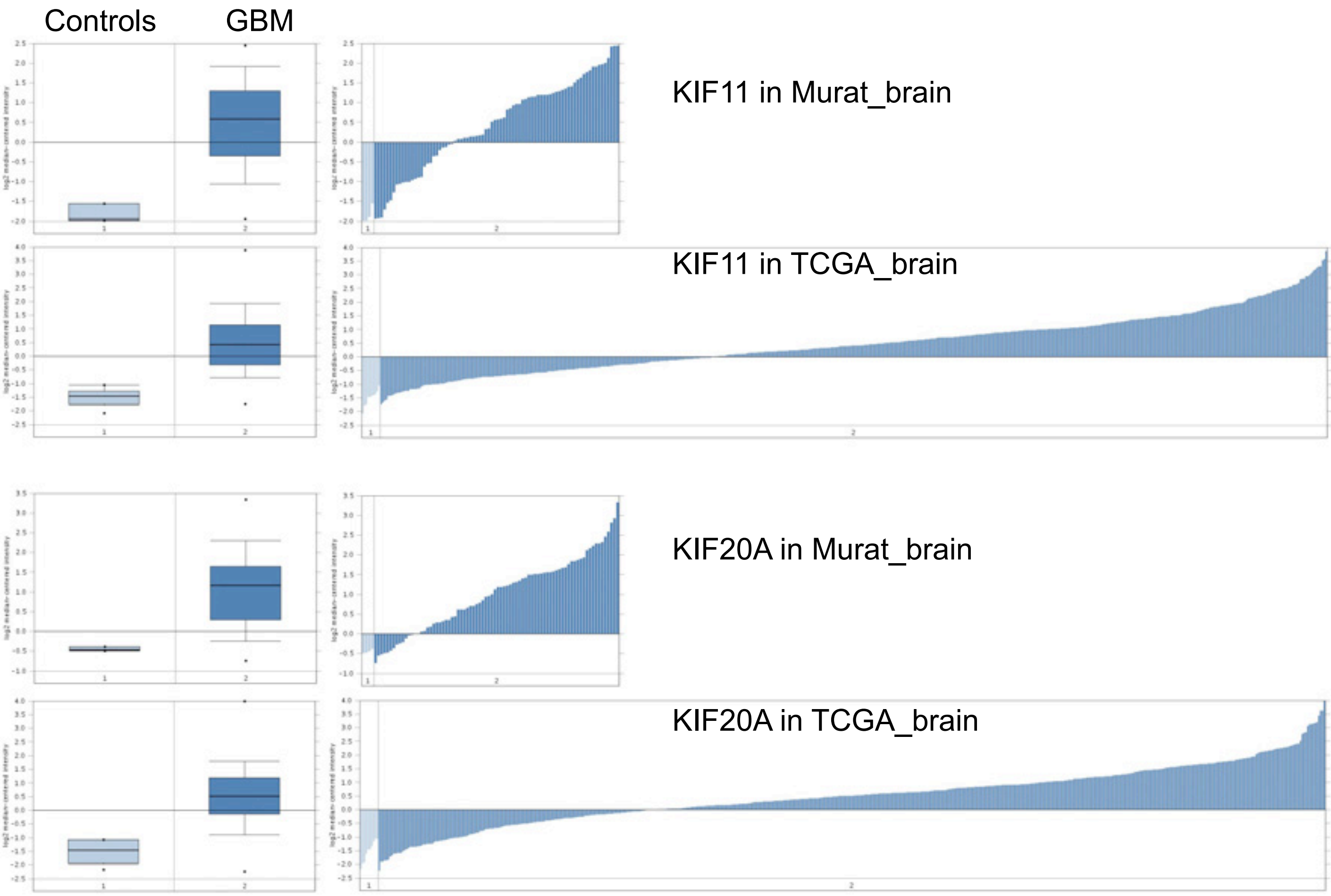

Even though over expression of KIF11 and KIF20A seems obvious from box plot presentation, visualization of the whole dataset (expression per patient) shows heterogeneity of expression in two independent glioblastoma studies (Murat_brain: 4 controls, 80 GBMs; TCGA_brain: 10 controls, 515 GBMs) with approximately two-thirds of patients over expressing.

Figure S4

Vascular expression of KIF20A/Mklp2 in normal tissues and glioblastoma

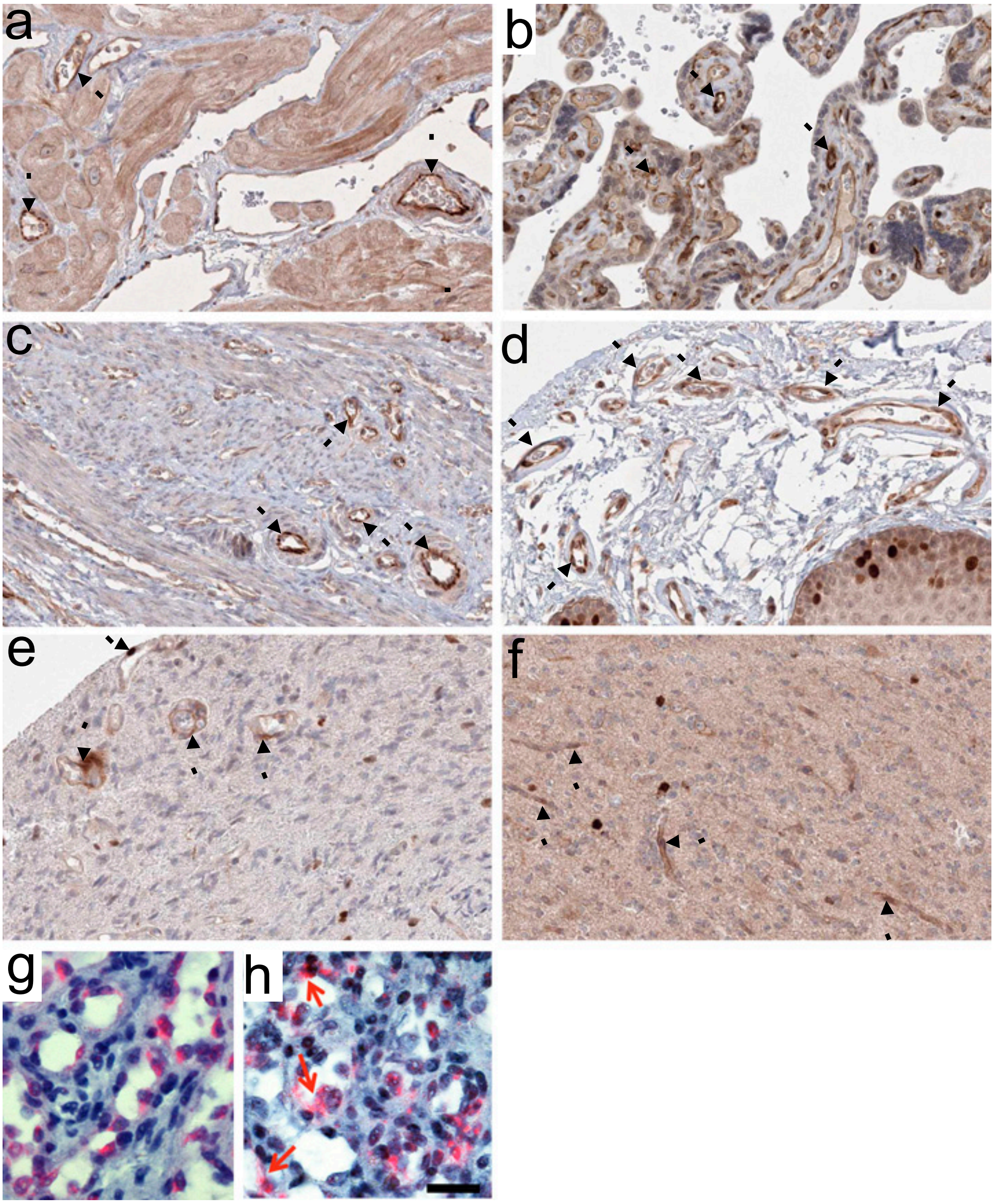

Mklp2 protein is expressed with varying intensities in endothelial cells (black arrows) in several normal tissues: (a) heart, (b) placenta, (c) endometrium and oral mucosa (d). In glioblastoma (e, f) a diffuse background signal is found in all cells, but several tumor capillaries can be recognized by stronger Mklp2 expression in the endothelium (black arrows). (g) Endothelial cells in tumor capillaries can be identified by in situ hybridization using a PECAM1 probe (in red). (h) KIF20A transcripts exhibit a very similar expression pattern in tumor capillaries in glioblastoma (in red, arrows point to endothelial cells). Serial sections are shown (a-f: bar = 30 μm; g, h: bar = 30 μm).

Figure S5

Co-expression of VEGF-induced kinesins in brain tumors

a

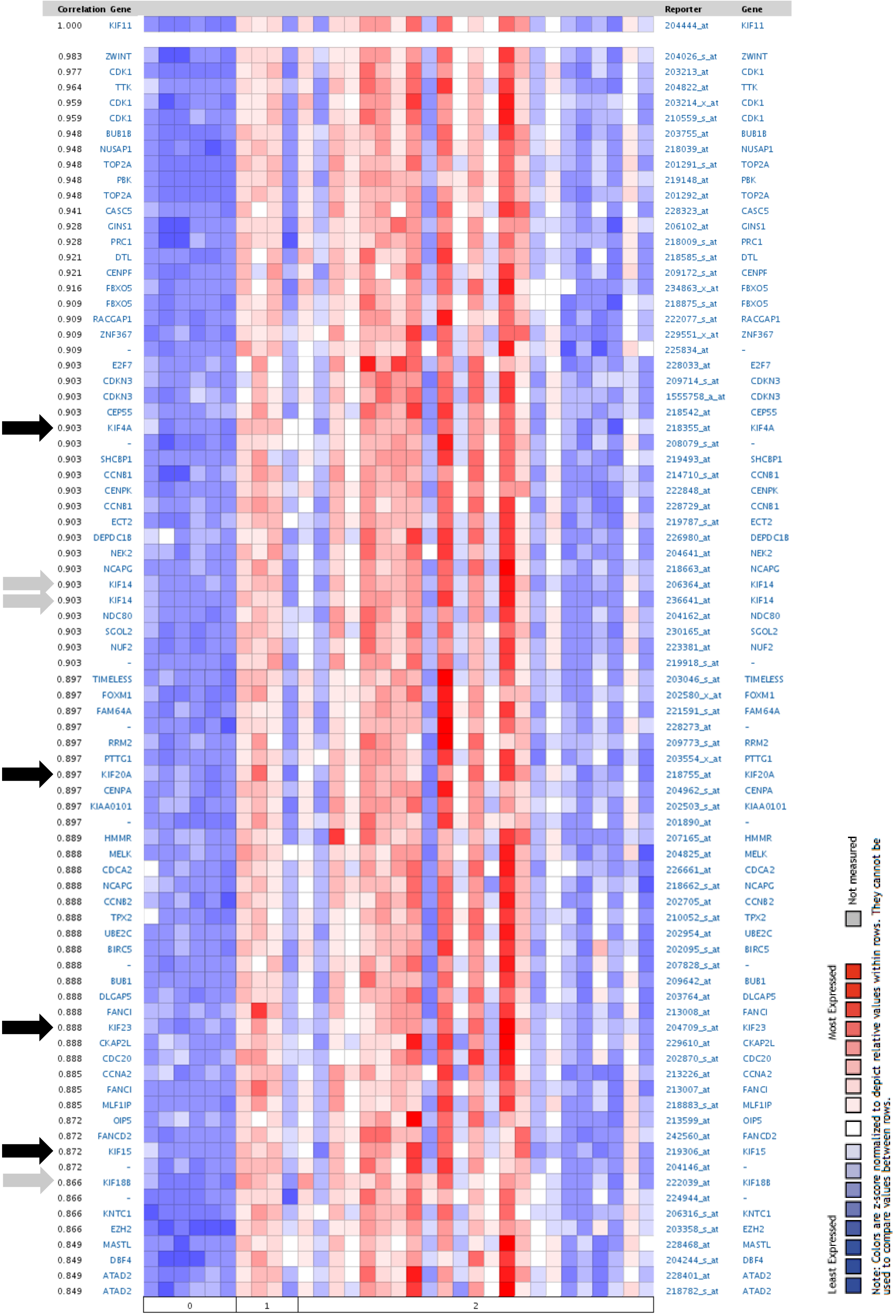

Figure S5 (cont'd)

Co-expression of VEGF-induced kinesins in brain tumors

(a) French_brain study. Note that all kinesins induced by VEGF are highly co-expressed (KIF11 as a bait), with correlation values ranging from 0.903 for KIF4A to 0.872 for KIF15 (black arrows). Other kinesins such as KIF14 and KIF18B also show co-regulation (grey arrows). Legend: 0: Normal brain (n=6), 1: Anaplastic Oligoastrocytoma (n=4), 2: Anaplastic Oligodendroglioma (n=23).

Figure S5 (cont'd)

Co-expression of VEGF-induced kinesins in brain tumors

b

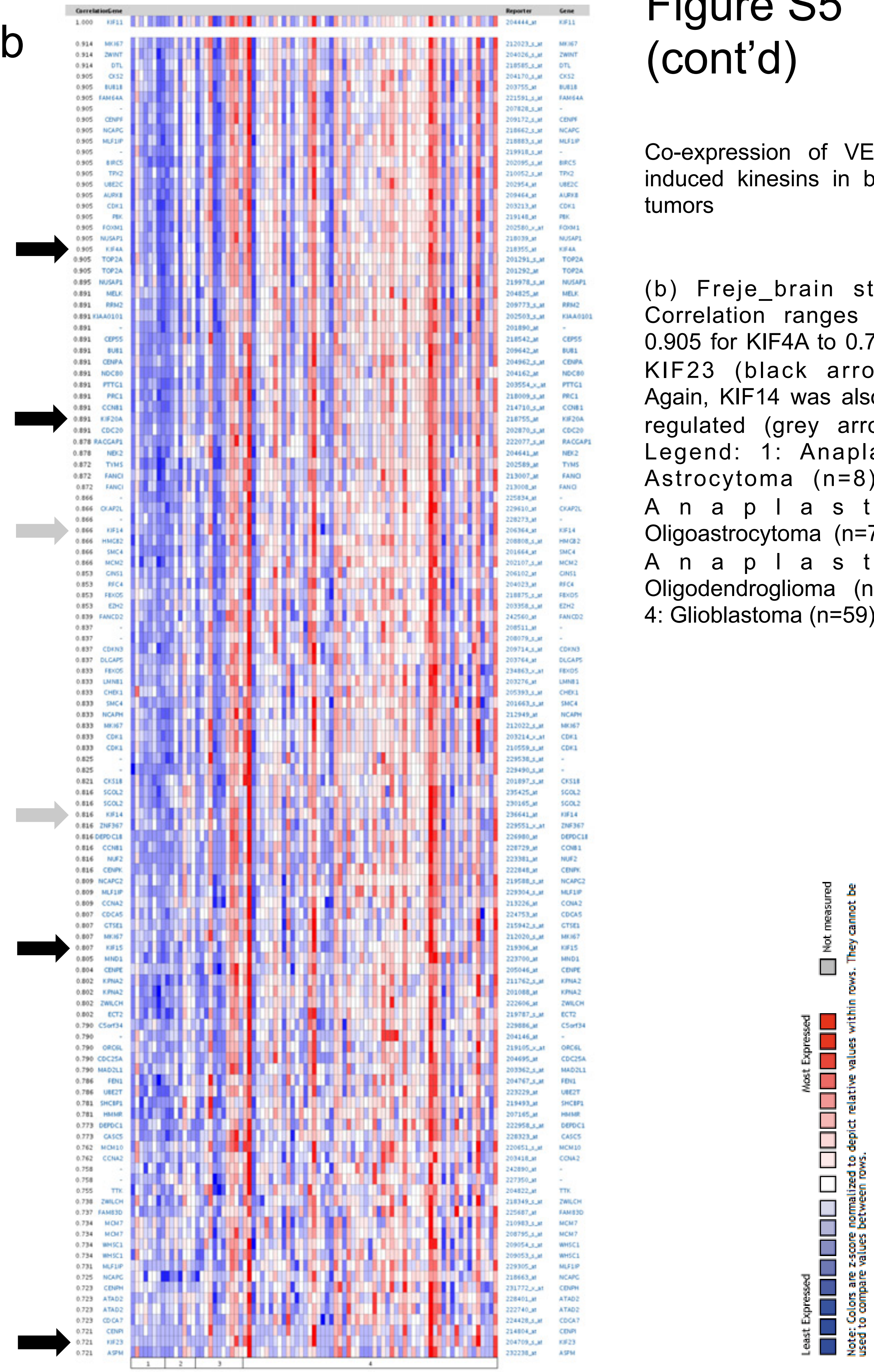

(b) Freje_brain study: Correlation ranges from 0.905 for KIF4A to 0.71 for KIF23 (black arrows). Again, KIF14 was also co-regulated (grey arrows). Legend: 1: Anaplastic Astrocytoma (n=8), 2: Anaplastic Oligoastrocytoma (n=7), 3: Anaplastic Oligodendroglioma (n=11), 4: Glioblastoma (n=59).

Figure S6

Expression of VEGF-induced kinesins depends on the vascularization status in hepatocellular carcinoma (HCC)

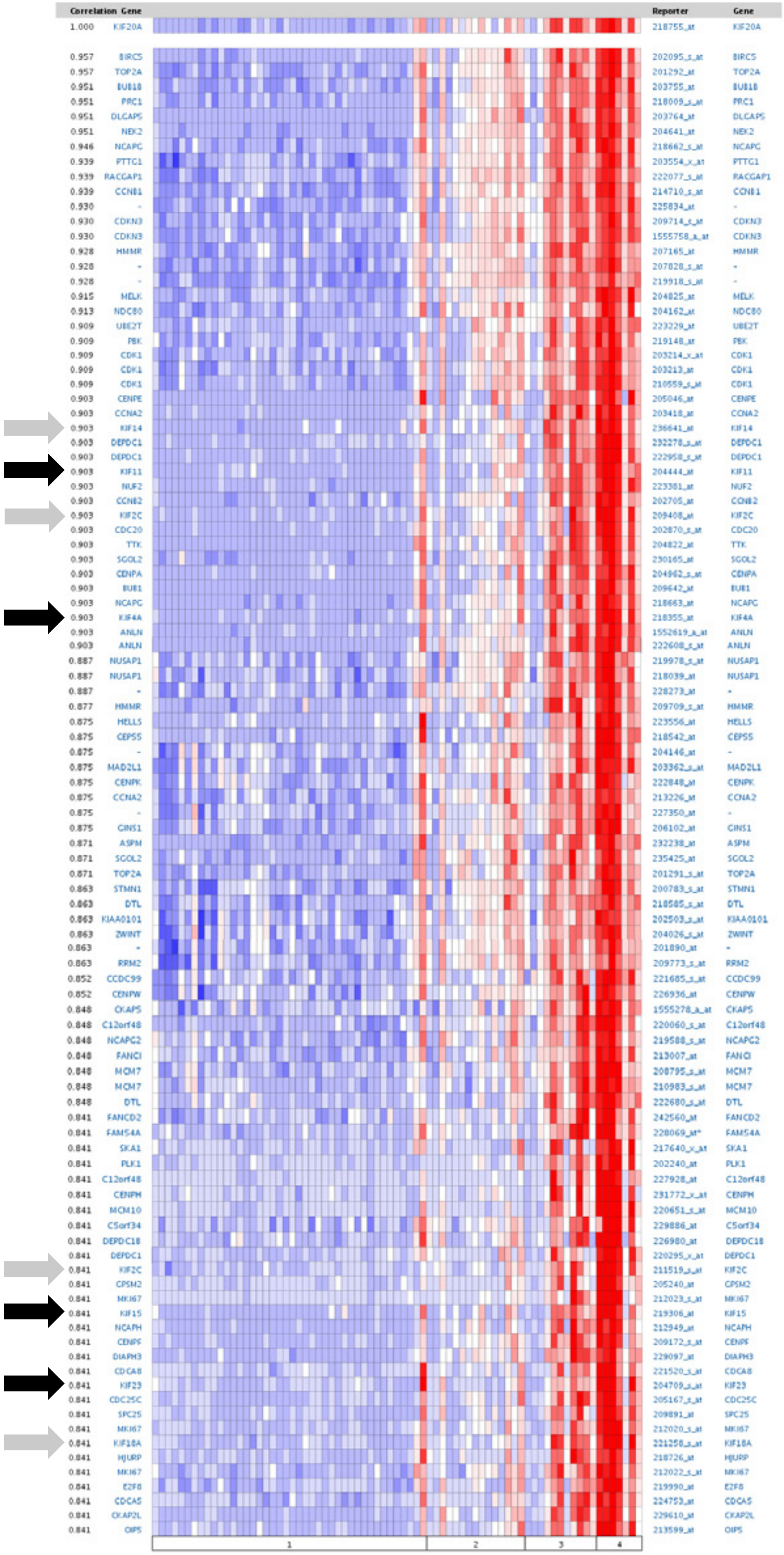

KIF11 (0.903), KIF4A (0.903) and KIF15 (0.841) expression correlates with KIF20A expression (data from the Wurmbach_liver study). Notably, these kinesins are over expressed in highly vascularized HCC samples, compared to controls.

Legend: 0: normal liver (n=42), 1: No visible vascular invasion (n=15), 2: microscopic vascular invasion (n=11); macroscopic vascular invasion (n=7).

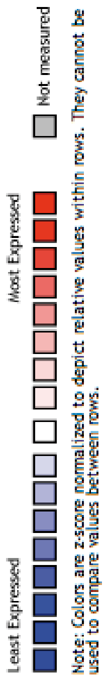

Figure S7

Relative expression levels of KIF11 and KIF20A transcripts in 84 human tissus and cell lines

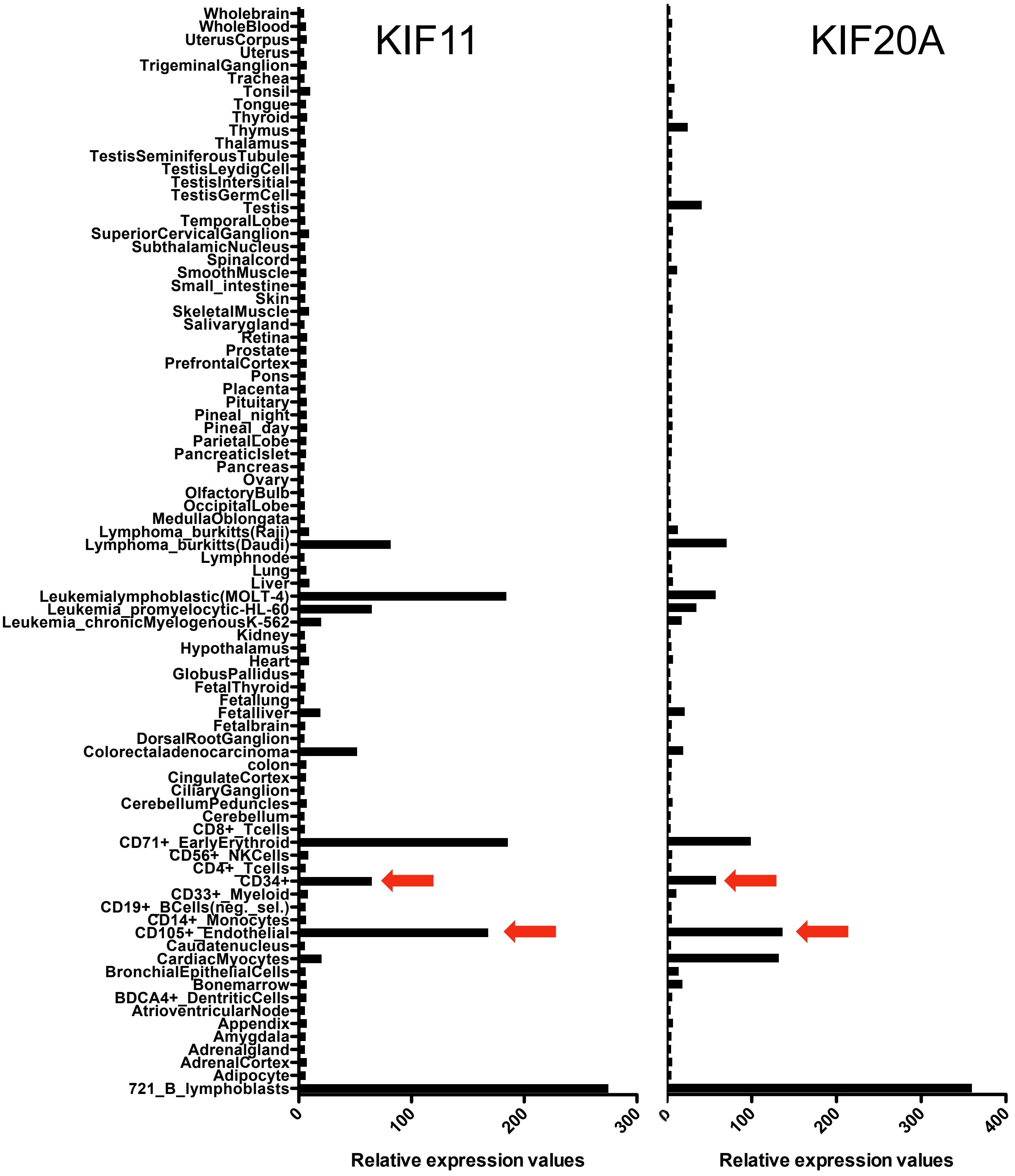

High levels of KIF11 and KIF20A transcripts were essentially found in malignant lymphoma cells and CD105+ / CD34+ endothelial cells (red arrows). There is a high correlation of KIF11 and KIF20A expression levels throughout all tissues (Spearman r=0.84, P<0.0001). Data were obtained using the BioGPS project.

Figure S8

Cell proliferation is a rare occurrence in the tubulogenesis assay

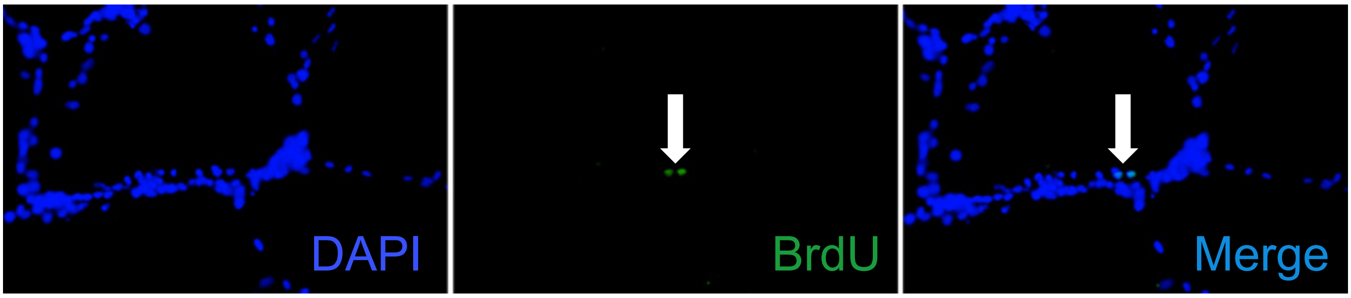

To exclude that endothelial cell proliferation occurs in our assay conditions, cells were incubated with BrdU and allowed to form tubes on matrigel for 14h30. Anti-BrdU antibody (in green) rarely detected BrdU signal in cells, one example is shown (arrows).

Figure S9

Eg5 inhibition impairs endothelial cell adhesion and spreading

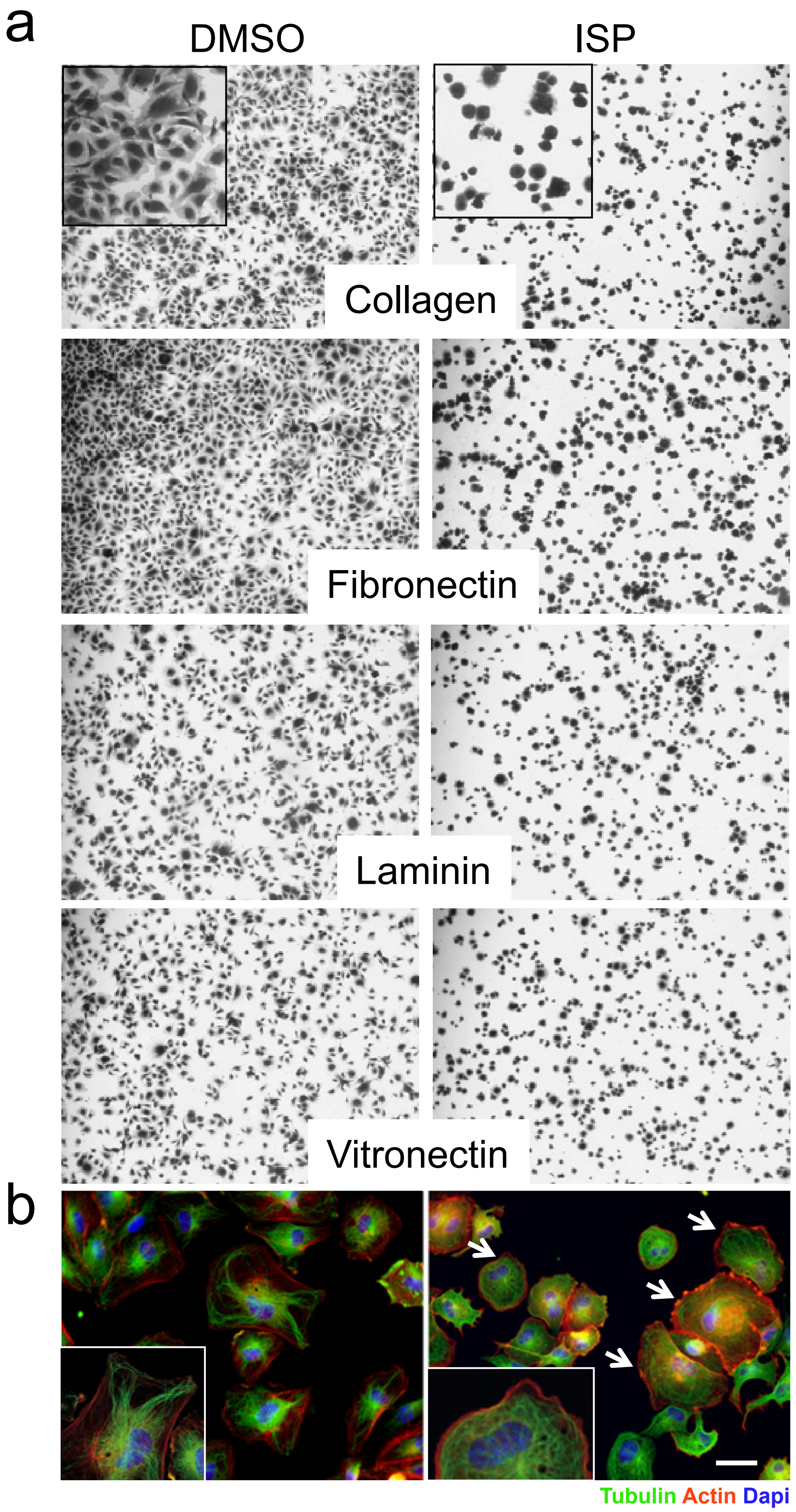

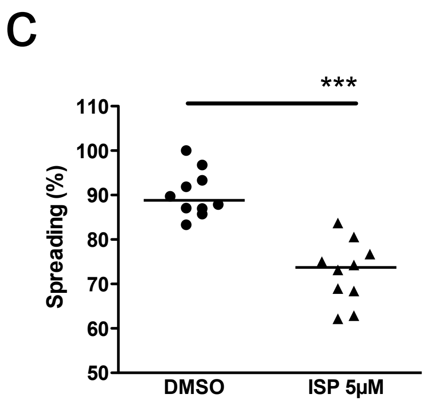

a) Inhibition of Eg5 by ispinesib reduces cell adhesion of endothelial cells plated on indicated matrix proteins b) Reorganization of the actin cytoskeleton: appearance of cortical actin and strong reduction of stress fibers (arrows and insert; bar in b=50μM) c) Cell spreading is reduced significantly after Eg5 blockade (p>0.0001).

Figure S10

Kinesin inhibition impairs migration of HUVECs

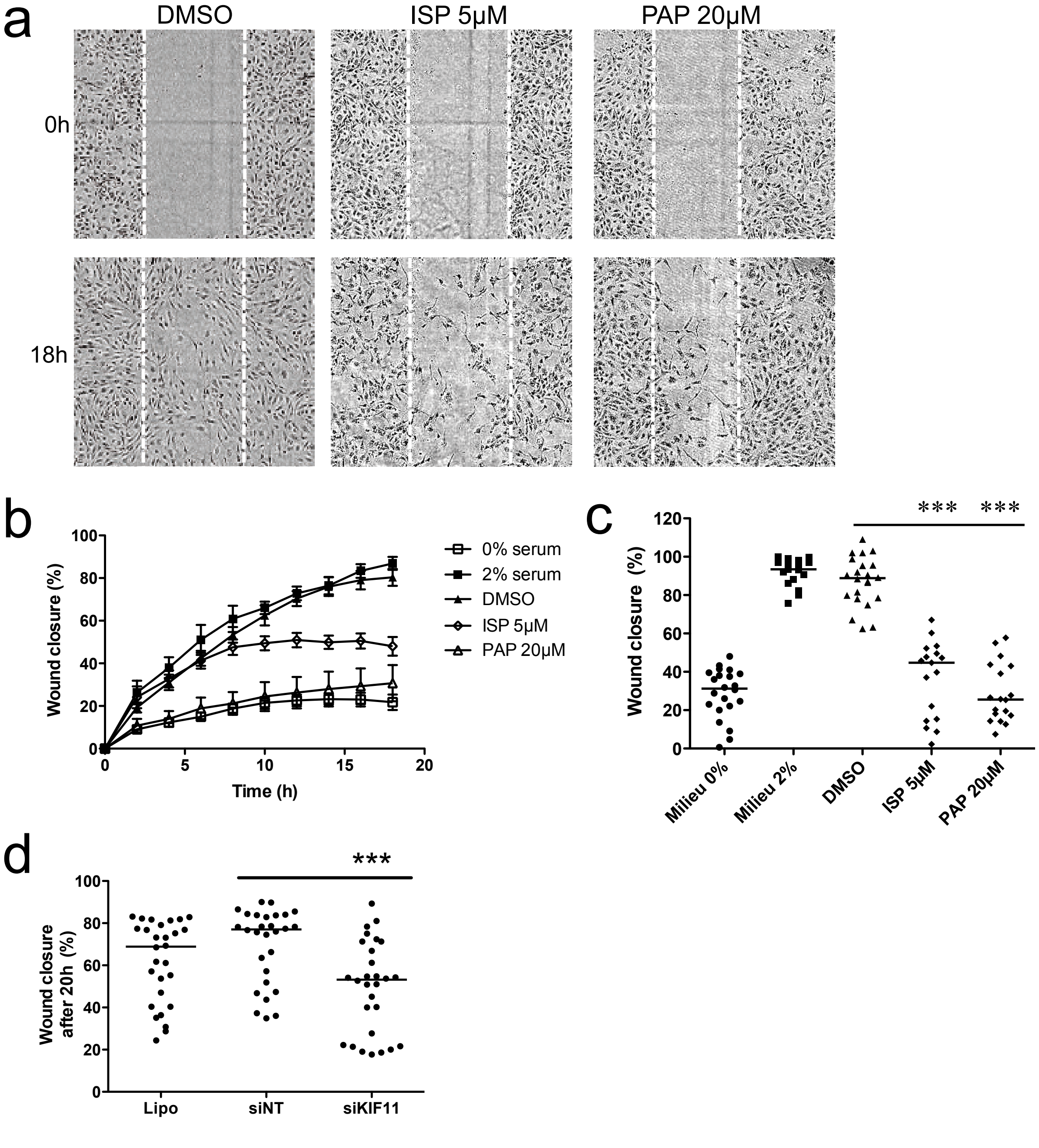

a) Representative pictures of HUVEC migration treated with indicated drugs at 0h and 18h after wounding. b) Quantification of cell migration into the wounded area over time. Percentage is indicated as mean+/- SEM. Note strong inhibition of EC migration by ISP and PAP. c) For statistical analysis, four experiments were pooled and percentages of wound closure at the last time point were compared. ISP and PAP significantly block EC migration (P<0.0001). d) siRNA-mediated knock down of KIF11 also led to a significant reduction of endothelial cell migration (P=0.0007).

Figure S11

Eg5 and Mklp2 blockade inhibits angiogenic sprouting in the mouse aortic ring assay

a

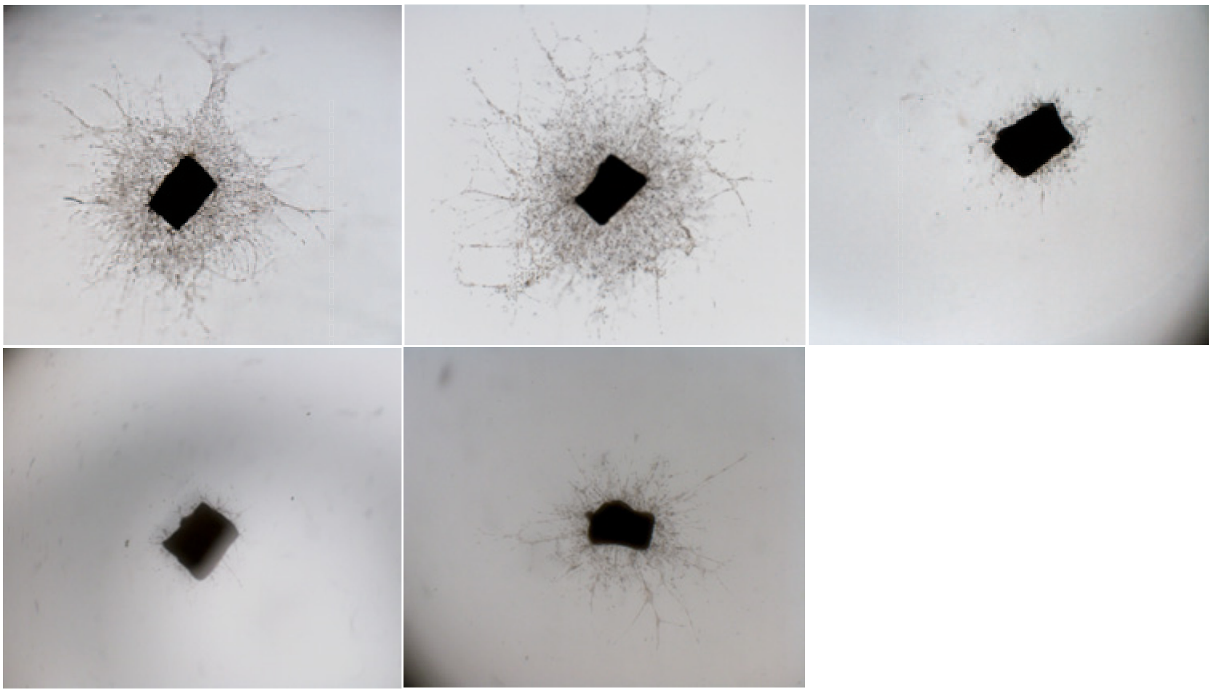

b

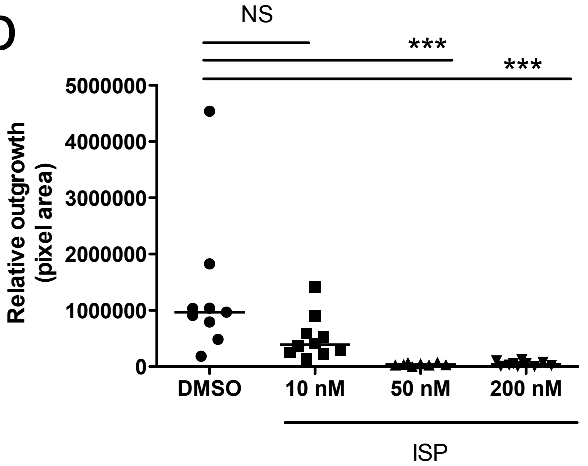

c

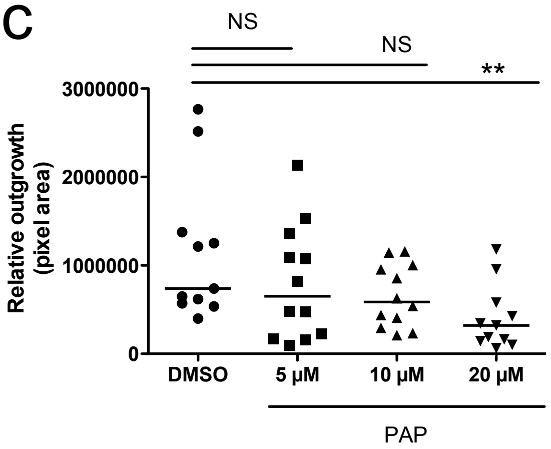

a) Representative pictures of mouse aortic rings in culture after 8 days. b) Quantification of cellular outgrowth around the aortic rings revealed significant reduction at doses around 50 nm for ispinesib and 20 μM for paprotrain.

Figure S12

a Fluorescence *in situ* hybridization; 24 hpf

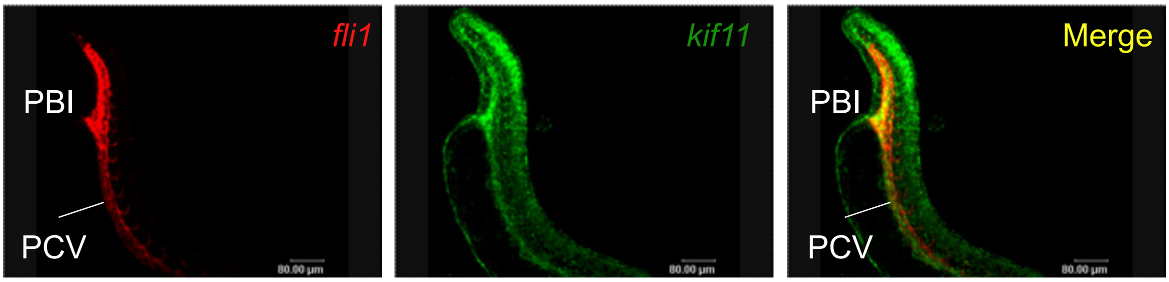

b Vascular defects in *kif11* Tg(kdrl:EGFP)[s843] morphants (48 hpf)

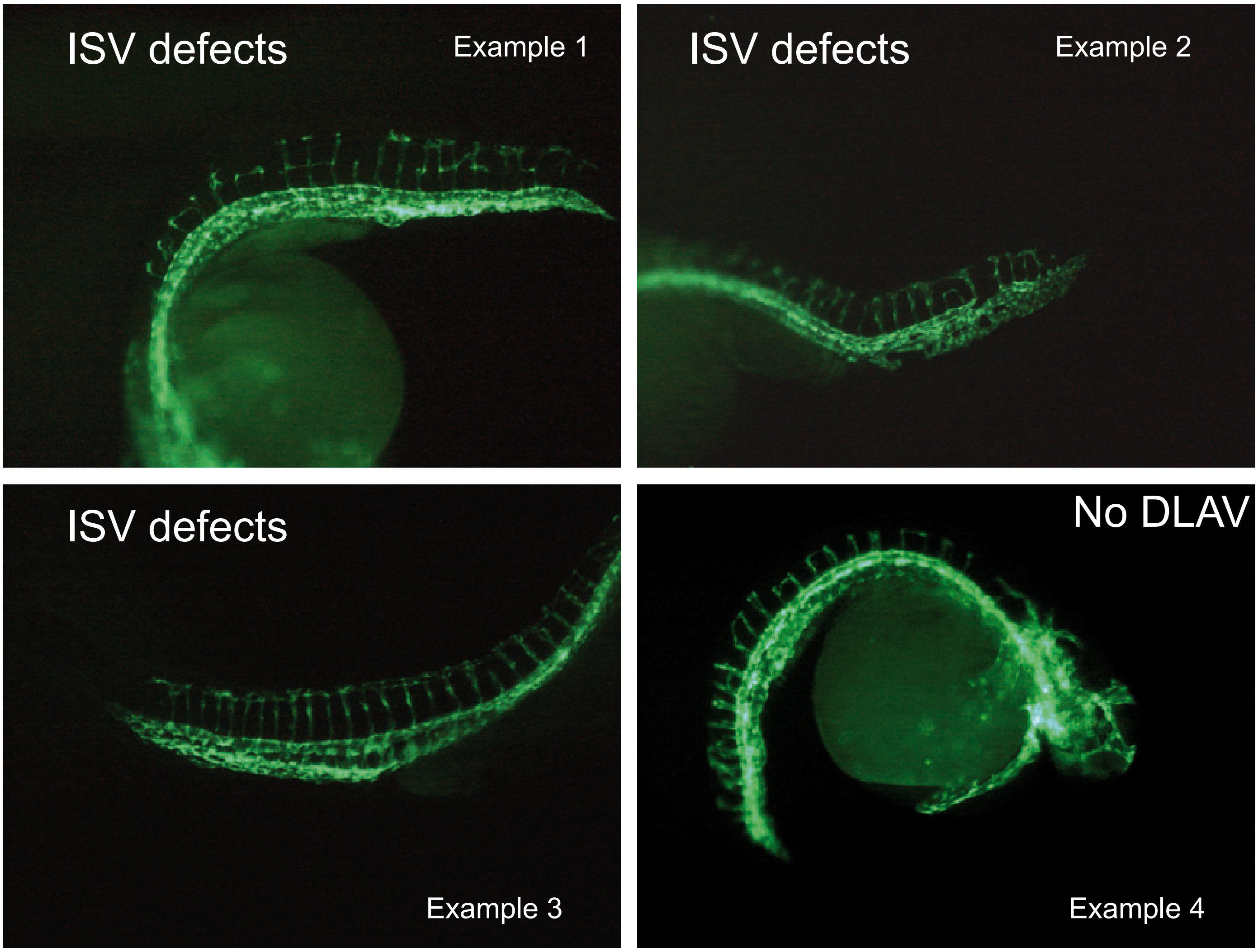

a) Zebrafish embryos at 24 hpf were hybridized with probes for the vascular/hematopoietic reporter transcript *fli1* (in red) or *kif11* mRNA (in green). Note that co-localisation occurs in the PBI and PCV. b) *kif11* knock-down embryos exhibited variable degrees of vascular malformation, mainly in the ISVs or the DLAV. Shown are four embryos with typical defects. Magnification x115.
