# Supplementary material for: Impaired angiogenesis and tumor development by inhibition of the mitotic kinesin Eg5
Source: Oncotarget. 2013 Oct 26;4(12):2302–16. doi: 10.18632/oncotarget.1490 (PMC3926828; doi:10.18632/oncotarget.1490)
Supplement: Supplementary file 3 [file oncotarget-04-2302-s003.doc]

**Supplemental Methods**

**Molecular biology experiments**

**Microarray hybridization**

Microarray experiments were carried out using standard Affymetrix protocols. To identify genes, which were regulated by VEGF-A with reasonable reproducibility, we applied the following selection criteria: a gene had to be regulated in at least two of the three comparisons more than 2-times and had to be found “present” in the VEGF-A group (if up-regulated by VEGF-A). 317 probe sets detected up regulated transcripts by VEGF-A using these criteria. Within this group were 80 probes, which were regulated more then 2-times in all three comparisons. To evidence consistency of VEGF-A-induced gene expression between the three different biological replicas compared to pooled control CAMs, we performed Spearman correlation analysis after log-transformation of fold-change expression. Original data sets are deposited at GEO (<https://www.ncbi.nlm.nih.gov/geo/>) with following identifiers: GSM364860, GSM364862, GSM364863, GSM364864.

**Bioinformatic analysis of microarray data**

To extract biological themes associated with VEGF-A-induced genes, we used Gene Ontology through the DAVID interface (<http://david.abcc.ncifcrf.gov/>).

To focus on human ortholog genes with preferential expression in the endothelium, we analyzed the 317 genes by a recently developed, validated approach. In brief, genes were submitted to an automated BLAST pipeline to assign orthologs. Only orthologs were then compared to endothelial and non-endothelial expressed sequence tags (ETS) pools, followed by FDR-based statistics. The ratio of the number of corresponding ESTs gives an estimate of EC or non-EC specifity. In a similar manner, all kinesins identifiable by a KIFxx gene symbol were analyzed by this method and EC or non-EC enrichment was assigned. For expression and co-regulation studies in human tumors, Oncomine™ (Compendia Bioscience, Ann Arbor, MI) was used for analysis and visualization. Data of the following studies were included in our analysis: Hou_Lung, Detwiller_sarcoma, Sun_brain, Murat_brain, TCGA_brain, French_brain, Freje_brain and Wurmbach_liver (Identification of datasets: Oncomine nomenclature, references about each study are on http://oncomine.com).

**Real-time RT-PCR (microarray verification)**

mRNA used for microarray hybridization was used to verify fold-changes of indicated genes. 10 µl SYBR Green (ABGene) were mixed with 6 µl ddH2O and 2 µl primers and 2µl cDNA diluted 1/5^th^. 42 cycles were run on a Stragene thermocycler. Fold-change was calculated using the ^ΔΔ^Ct-method. Validated chick-specific primer sequences were as followed:

| KIF4A | F | AGGGTGGTTTTGTCCCCTAC |
| --- | --- | --- |
|  | R | GGGCTTACACAGGCAATCAT |
| KIF11 | F | GGAACAACTGAGGCAGAAGC |
|  | R | AGCAGGTCCTCATCTGCATT |
| KIF15 | F | TATCGGGATTCCAAGCTCAC |
|  | R | TGGATCCTGGATGGACATTT |
| KIF20A | F | CAGCCAATTTACCAGCCATT |
|  | R | CTCCTGTGGTGCTGAGTGAA |
| KIF23 | F | CAACGTCAGGCTTCTGACAA |
|  | R | CAGCTGCTTTGCTGCTACAC |
| HNRPH1 | F | GATTTCGGTCTTGGTGTTCG |
|  | R | TAACTCGTGGCAGACACAGC |
|  |  |  |

**KIF11 siRNA knock down**

HUVECs (20.000 cellules/100µL) were seeded in 96-well plates (Essen Bioscience). After 5h they were transfected overnight with 50nM of siRNA KIF11 (Reference HSS105842, Fisher Scientific, Illkirch) or non-target siRNA using RNAimax as indicated by the manufacturer. Transfection medium was replaced by 1% normal medium and the scratch wound assay was performed as described below in the cell culture section. Migration data after 20h from 4 independent experiments were pooled and efficacy of siRNA KIF11 vs non-target siRNA was evaluated using Mann-Whitneys U-test.

**Cell culture experiments**

**Cell cultures**

hCMEC/D3 cells were a kind gift of Dr. P. O. Couraud (Institut Cochin, Paris) and cultured as described. HUVECs were purchased (Promocell) and cultured as indicated by the manufacturer or isolated freshly from healthy donors using standard protocols. LECs were isolated from 2 healthy donors according to a published protocol. Tumor cells were maintained in DMEM (Invitrogen, Cergy Pointoise Cedex, France) with 10% fetal bovine serum, antibiotics and L-glutamine.

**Proliferation assays (time course)**

Cells (HUVEcs, LECs and fibroblasts) were harvested, re-suspended in culture medium to a concentration of 10^4^ cells/100 µl and seeded into wells of 96-well culture plates (100 µl/per well). Neuroblastoma cell-lines (IMR5, SHEP, SKIN, Chp134, Gimen, Kelly) were used in a concentration of 5x10^3^ cells/200µl. For each condition and time point, 6-8 replicate wells were measured. At indicated time points, cells were fixed with 5% glutaraldehyde. After incubation for 20 minutes, the glutaraldehyde-containing medium was removed and plates were washed twice with deionized water. The plates were allowed to dry and 100µl crystal violet solution (0.1% in deionized water) was added to each well. After 20 minutes, the dye was removed; the plates were washed thrice with deionized water and dried again. The dye was resolved in 100 µl of 10% acetic acid and absorption at 570 nm was determined using a micro plate reader (Molecular Devices, Sunnyvale, CA).

**Proliferation assays (at 72h)**

Cells were seeded after 6 h serum starvation in 500 μl full culture medium at indicated densities in 24-well cell culture flasks (LEC: 2.5x10^4^, HUAEC: 2.5x10^4^, HUVEC: 2x10^4^, hCMEC/D3: 2x10^4^, BAE: 2x10^4^, U87: 1.5x10^4^, T98G: 1x10^4^, Gl261: 7.5x10^3^. Cells were treated with inhibitor at indicated doses and after 72h, cells were trypsinized and counted on a Beckman Coulter counter. DMSO at the highest inhibitor concentration served as control treatment.

**Adhesion assays**

HUVEC were pre-treated for 14h with solvent or Eg5 or Mklp2 inhibitors at indicated concentrations. 8000 HUVECs were seeded on 96-well culture dishes coated with indicated ECM proteins: collagen (100µg/ml BD Biosciences), fibronectin (0.5µg/ml, Sigma), laminin (5µg/ml, Sigma) and vitronectin (0.5µg/ml, Sigma). One hour later, cells were washed, stained with Coomassie Blue and photographed at 20x magnification. Statistical analysis of the percentage of normally spread cells was done using the Krusal-Wallis test followed by Dunn’s multiple comparison test.

**Scratch wound assay**

HUVECs (2.5x10^4^ cells/well) were seeded in 96-well plates (Essen Imagelock™; essenbioscence.com) coated with collagen (BD Biosciences) and allowed to adhere for 6 hours. Confluent cells were starved overnight in Endothelial Cell Growth Medium (Promocell, Heidelberg, Germany) without growth factors and serum. The 96-pin WoundMaker (Essen) was used to create simultaneously precise and reproducible wounds in all wells. Non-adherent cells are removed with reduced medium. HUVEC were treated with inhibitors at indicated concentrations and the plate was placed in IncuCyte™. Wound images were acquired automatically at 2 hours intervals. Statistical analysis of the wound closure area was performed at the 18h time point using the Krusal-Wallis test followed by Dunn’s multiple comparison test.

**In vitro angiogenesis**

4000 HUVECs were placed in μ-slide angiogenesis tissue culture plates coated with growth factor-reduced Matrigel (Ibidi, Biovalley, France) and covered with medium containing solvent or different inhibitors. After 14h, photos were taken at 4-fold magnification using an inverted light microscope. Digital images were uploaded to the WIMASIS image analysis platform and analyzed in an automated fashion (http://wimasis.com). The parameters “number of chords”, “number of loops”, “isolated chords” and “branching points” were determined from two independent experiments and compared using the Kruskal-Wallis test followed by Dunn’s post-test. For videomicroscopy, cultures were placed at controlled cell culture conditions under a motorized Nikon Ti-E Inverted MEA53100 Microscope coupled to a Hamamatsu ORCA-R2 camera and photos were taken every ten minutes over a 14h time period. Indirubin-3’-monoxime (IRO), which has been shown to inhibit angiogenesis in zebrafish embryos as well as tube formation, was used as positive control for evaluating the results with the automated blinded WIMASIS tube formation analysis platform. IRO treatment significantly reduced the number of branching points (45.4% of controls, P=0.0003), increased the number of independent (fragmented) chords (215.2% over controls, P=0.0004) and reduced loop formation (30.9% of controls, P=0.0077).

**Mouse aortic ring assays**

C57Bl6 mice were sacrificed by cervical dislocation. Aortas were isolated and transferred to petri dishes containing complete endothelial basal medium (EBM2, Promocell) with antibiotics. Connective tissue and fat tissue surrounding the vessel was carefully omitted and rings were cut using a scalpel. Rings were placed in 48-well dishes, covered with growth factor-reduced matrigel (BD Bioscience) and after polymerization at 37°C, 300μl EBM2 medium were added per well. Medium with drugs is replaced every two days, starting from the third day of incubation. Photos were taking using a macroscope (Nikon AZ 100M) and vascular outgrowth area was quantified using Image J software (http://rsbweb.nih.gov/ij/). Statistical analysis of treated vs. control cultures were performed at the 8^th^ day after first treatment.

**Histology, in situ hybridizations and Western Blots**

**Immunofluorescence (cell spreading assay)**

HUVECs are seeded for 1h on coverslips coated with 100µg/ml of collagen (BD Biosciences). After rinsing with PBS fixation with PAF 4% they were permeabilized with PBS-triton, blocked with BSA and incubated with mouse monoclonal anti α-tubulin (1:1000, Sigma T5168) for 2h at room temperature. Cells are rinsed and incubated with goat anti mouse antibody coupled with Alexa 488 (Molecular Probes), phalloidin-tetramethylrhodamine B isothiocyanate conjugate (1:1000, P1951 Sigma) and counterstained with DAPI (Invitrogen).

**Immunofluorescence (in vitro angiogenesis assays)**

In vitro angiogenesis cultures are directly fixed by adding 10µL of PAF 4% to each well and rinsed with PBS. Cell nuclei are stained with DAPI and cultures are mounted in fluoromounting medium (Electron Microscopy Science). For assessment of cell proliferation during tubulogenesis, HUVEC are treated for 14h with BrdU (Invitrogen) at 1µM and seeded on reduced matrigel. BrDu incorporation is revealed with a FITC-coupled monoclonal anti-BrdU antibody (Becton Dickinson) and cultures are mounted as described above.

**Immunofluorescence (human foreskin)**

Foreskin of 2 – 3 year-old boys (3 specimens) were taken directly from the operating room. Specimens were rinsed in PBS, incubated in 15% and 30% sucrose and embedded in tissue freeze medium (Neg50; Richard-Allan Scientific, US) without any fixation. Cryosections of 16 - 20 µm thickness were prepared. Non-specific binding of antibodies was blocked with 1% bovine serum albumin (BSA) for 10 min. Primary antibodies were rabbit-anti-human CD31 (1:100; Abcam, Cambridge, UK), mouse-anti-human podoplanin (1:100; ReliaTech, Wolfenbüttel, Germany) and rabbit-ant-human Eg5 (1:500; Abcam ab37009). Secondary antibodies (1:200) were Alexa 588-conjugated donkey-anti-rabbit and Alexa 594-conjugated goat-anti-mouse (Molecular Probes / Invitrogen, Darmstadt, Germany). Nuclei were counter-stained with 4′,6-diamidino-2-phenylindole (DAPI).

**Immunodetection of Eg5 and Mklp2 (human protein atlas)**

We queried the protein atlas for expression of Eg5 and Mklp2. Images were retrieved from the following protein atlas samples: glioblastoma (patient ID 3092, Eg5 antibody HPA006916; patient ID 2726, Eg5 antibody HPA010568; patient ID 1537, Eg5 antibody CAB017617; ID 3022 and 2527, Mklp2 antibody HPA36909), renal cell carcinoma (patient ID 3061, antibody Eg5 HPA006916; patient ID 1722, Eg5 antibody CAB017617, patient ID 1901, Eg5 antibody CAB017617), normal heart (patient ID 2278, Mklp2 antibody HPA036909), placenta (patient ID 2515, Mklp2 antibody HPA036909), endometrium (patient ID 2175, Mklp2 antibody HPA036909) and oral mucosa (patient ID 1505, Mklp2 antibody HPA036909). Original images were downloaded and assembled in Adobe Photoshop CS4.

**Evaluation of vascular parameters after Eg5 inhibition using CD31 immunoreactivity (RENCA tumor model)**

Vessel density was evaluated on frozen section of RENCA tumors. Primary antibody was anti-CD31 (1:100; Pharmigen), secondary antibody (1:400; goat anti-rabbit Alexa 488, Interchim) and sections were mounted in DAPI-containing mounting medium (Fluoroprobes). Given the heterogeneous nature of tumor angiogenesis in this model, we relied on a qualitative quantification method. In brief, one to five sections per tumor (ISP, n=16 tumors, controls; n=14 tumors) were photographed at 20x magnification using the same acquisition parameters (Nikon NIS Elements software). Digital images were opened in Adobe Photoshop CS4 and overall vascularity was classified per field from 0 to 3 by an experienced investigator (Dr Patrick Auguste, University Bordeaux) in a blinded fashion. The scores of the two groups were compared using the Mann Whitneys U-test (GraphPad Prism). To further characterize Eg5 inhibition effects on tumor vasculature, we treated mice bearing RENCA tumors with below-mentioned doses of ispinesib (n=4) or solvent (n=4) on day 11 and 12 after tumor cell injection and harvested tumors 24h later for histological analysis. For vessel diameter measurements, the visible interior surface of capillaries in five sections per tumor was determined using NIS elements software. An arbitrary cut off value of 300 square pixels was applied and number of capillaries above that value counted in the two groups and evaluated using the Mann-Whitneys U-test. The tumor mouse model protocol was authorized by the animal ethics commission of Bordeaux (No.: 50120003A).

**In situ hybridization (human tumor sections)**

In situ hybridization using QuantiViewRNA reagents was performed as recommended by the supplier (Panomics-Affymetrix). The use of branched DNA signal amplification is expected to provide up to a 500-fold amplification for low abundance mRNAs. Probe sets were designed by Panomics-Affymetrix from human CD31 NM_000442 or KIF20A NM_005733. Each probe set consists of 10 or more nucleotide sequence pairs. The oligonucleotide probe pairs are designed to hybridize to adjacent segments on the target RNA allowing the hybridization of a preamplification probe, which spans the hybridized probe pair. This signal is further amplified by bDNA and detected by label probes.

Formalin fixed paraffin embedded human glioblastoma biopsies were provided by the Regional University Hospital of Bordeaux. Five μm sections were collected on positively charged Superfrost slides and dried at 60°C for 30 min, then post fixed in 10% formaldehyde for 1 hour at room temperature. Slides were washed briefly in PBS twice before being air-dried. For deparaffinization, slides were incubated at 80°C for 3 minutes followed by submersion in 200 mL of Histo-Clear® reagent (National Diagnostics, USA) for 10 minutes at room temperature with frequent shaking. Residual Histo-Clear reagent was removed by washing in 95 percent ethanol. Sections were circled with a Hydrophobic Pen (Vector Laboratories, USA) then subjected to boiling pretreatment (Panomics) for 10 min. Slides were submerged in distilled water twice, then incubated with a protease solution (Panomics) at 40°C for 10 min. After three washes, the slides were transferred into 4% formaldehyde for 5 minutes followed by rinsing once in 1x PBS. Preamplification and amplification steps were performed following Panomics-Affymetrix instructions. After incubation with the label probe, slides were washed and 200 µL of the AP-enhancer solution was added to the section and incubated at room temperature for 5 to 10 minutes. Fast Red was added to the tissue section for 30 minutes at 40°C in the dark. Slides were then rinsed in 1x PBS. To fix, slides were incubated in 4% formaldehyde for 5 minutes at room temperature then washed in 1x PBS and counterstained with Gill’s hematoxylin before being mounted using Dako®Ultramount medium (Dako, USA). Results were viewed under bright field for CD31 (high rate expression per endothelial cell) or epifluorescence for KIF20A (low abundance mRNA per cell).

**Fluorescent whole mount *in situ* hybridizations and imaging (zebrafish embryos)**

Experiments were carried out as described, with minor modifications . Double fluorescent *in situ* hybridizations have been performed as described . The different antisense riboprobes were labeled either with digoxigenin labeling mix (Roche) or DNP-11-UTP ribonucleotides (Roche). Hybridizations were subsequently performed using the TSAi Plus system kit (Perkin Elmer). Briefly, after permeabilization, hybridization and washes, embryos were blocked in TNT buffer with 0.5% Blocking Reagent, and then with an anti-DNP peroxidase-conjugated pre-absorbed antibody (1:200). Revelation was sequential: first, the green color was developed with tyramide-FITC. Then, after inactivation of the first antibody with H_2_O_2_, the embryos were incubated with an anti-DIG peroxidase-conjugated preabsorbed antibody (1:200) and the red colour was developed with tyramide-Cy3. Embryos were then extensively washed in TNT buffer and then refixed in 1% paraformaldehyde until analysis. Antisense riboprobes were made by transcribing linearized cDNA clones with SP6, T7, or T3 polymerase using digoxigenin (DIG) or DNP labelling mix (Roche), according to manufacturer's instructions. They were subsequently purified on NucAway spin columns (Ambion) and ethanol-precipitated. The *kif11* probe was cloned from zebrafish embryos using the PCR2.1-TOPO cloning kit (Invitrogen) using the primers 5-AAGGAGCAGAGCCAAAAACA and 5-TAGCTTCATCGCACCACTTG according to the manufacturers indications. The *fli1* probe was synthesized as described.

Confocal imaging was performed using a Leica TCS SP2 confocal microscope. Digitized images were acquired using a DRY HC PL FLUOTAR 10× (NA 0.3) at 1024 × 1024 pixel resolution.  For multicolor imaging, FITC was excited with a 488-nm laser line and the emission light was recorded between 500 to 535 nm. Cy3 was excited with a 561-nm laser line and the emission light was recorded between 570 to 715 nm. Sequential scan acquisition mode between lines was used to avoid cross talk and a 2-frames average mode was performed to reduce background noise. Series of 20 optical sections were carried out to analyze the spatial distribution of fluorescence, and for each embryo, they were recorded with a Z-step at 1.3 μm (20x magnification) and 2.4 μm (10x magnification). Image processing, including maximum projection of Z-stacks, was performed with the Leica LCS software (version 2.5).

**Western Blot analysis**

Cells are treated with 10ng/mL of human recombinant VEGF-A (PeproTech France) in medium 0.2% SVF during 24h. Cells were solubilized at 1x10^7^ cells/mL in lysis buffer (1% NP-40, 20Mm Tris-HCl, pH 8.0, 137 mM NaCl, 10% glycerol, 2mM EDTA and protease inhibitors; Roche). The lysates were rocked gently at 4°C for 30 minutes then centrifuged at 14000 g for 5 minutes. The supernatants containing proteins are dosed using the Bradford assay. 50µg of protein from each treatment were deposed and detected by anti-Eg5 antibody (Abcam, Ab61199, 1:500). Membranes were scanned and pixel density quantified using a LI-COR Odyssey Infrared Imaging System. Control density was set to “1” for graphical presentation.

**Zebrafish experiments**

**Fish strains and maintenance**

Zebrafish (*Danio rerio*) kdr:EGFP strains were obtained from Zebrafish International Resource Center, Oregon University, USA (grant P40 RR12546 from the NIH-NCRR) and then reared by the GIGA Zebrafish Platform, Liege University, [http://www.giga.ulg.ac.be](http://www.giga.ulg.ac.be/), in recirculating system (Techniplast, Italy) at a maximal density of 7 fish/L. The water characteristics were as follows: pH = 7.4, conductivity = 500 µScm-1, temperature = 27°C. The light cycle was controlled (14h light, 10h dark). Fish were fed twice daily with dry powder (ZM Ltd, UK) with size adapted to the age, and once with fresh nauplii from *Artemia salina* (INVE Aquaculture, Belgium). Larvae aged less than 14 days were also fed twice daily with a live paramecia culture.

**Breeding**

The day before breeding, 2 males and 2 females were placed in breeding tanks out of the recirculating system, with an internal divider to prevent unwanted mating. On the day of breeding, fish were placed in fresh aquarium water and the divider was removed to allow mating. Eggs were collected every 30 minutes.

**Morpholino injection and phenotype analysis**

KIF11 zebrafish morpholino and control morpholino were purchased from GeneTools Inc. (Philomath, OR, USA) and used as recommended by manufacturer. Tg(kdrl:EGFP)^s843^ transgenic zebrafish were injected at the 1-2 cell stage with 0.2, 0.5 or 1 ng of KIF11 morpholino containing rhodamine dextran. Non-injected, damaged and non-fertilized embryos were sorted out directly after microinjection. At 24 hpf (hours post-fecundation) embryos were de-chorionated and treated with 1-phenyl 2-thiourea (PTU) to prevent pigmentation. At 48 hpf, embryos were anaesthetized using tricaine and phenotype-scored using the following criteria: class 1 = normal circulation, class 2 = normal circulation, affected tail and spasm, class 3 = affected circulation (reduced or absent) but heart beating, class 4 = dead or necrotic embryo. Embryos were analyzed using a Nikon SMZ 1500 and Leica MZ16F microscopes and images acquired by NIS Elements BR 2.30 or Leica Application suite 3.5.0 software.

**Ispinesib treatment of zebrafish embryos**

Normal zebrafish embryos (n=25 per dose; two independent experiments) were exposed to increasing doses of the specific Eg5 inhibitor ispinesib mesylate (0.1, 0.3, 1, 3 and 10 μM), from 24 to 48 hpf. Controls were treated with DMSO as used for the highest ispinesib dose. Dead embryos were counted; phenotype scoring of living embryos was done under a light microscope and blood flow and PBI (posterior blood island) morphology were scored as normal (=0) or reduced/modified (=1). As an additional morphological criterion number of blood cells was evaluated in a qualitative manner (0=normal, 1=reduced) and combined with the latter phenotype. Values of both experiments were pooled and analyzed using the Fishers Exact test with 99% interval of confidence.

**Orthotopic renal cell carcinoma model (RENCA model)**

10^5^ RENCA cells (mouse origin) were implanted in the subcapsular region of the left kidney of Balb/C mice (n=10 per group). Animals were observed and weighed twice weekly. Mice were treated with ispinesib mesylate (5 mg/kg) or solvent control (80% D5W, 10% ethanol, 10% Cremophore-El) twice weekly by subcutaneous injection. Animals were sacrificed when a >20% weigh loss occurred or when they presented other signs of suffering. Animals were sacrificed by cervical dislocation and the urogenital apparatus was prepared for tumor size and weight evaluation and CD31 immunohistochemistry. Comparison of tumor weight was done using the Mann Whitney’s U-test after pooling of two independent experiments.

**Supplemental References**

1. Huang da W, Sherman BT and Lempicki RA. Systematic and integrative analysis of large gene lists using DAVID bioinformatics resources. Nature protocols. 2009; 4(1):44-57.

2. Dennis G, Jr., Sherman BT, Hosack DA, Yang J, Gao W, Lane HC and Lempicki RA. DAVID: Database for Annotation, Visualization, and Integrated Discovery. Genome biology. 2003; 4(5):P3.

3. Javerzat S, Franco M, Herbert J, Platonova N, Peille AL, Pantesco V, De Vos J, Assou S, Bicknell R, Bikfalvi A and Hagedorn M. Correlating global gene regulation to angiogenesis in the developing chick extra-embryonic vascular system. PLoS One. 2009; 4(11):e7856.

4. Herbert JM, Stekel D, Sanderson S, Heath VL and Bicknell R. A novel method of differential gene expression analysis using multiple cDNA libraries applied to the identification of tumour endothelial genes. BMC Genomics. 2008; 9:153.

5. Weksler BB, Subileau EA, Perriere N, Charneau P, Holloway K, Leveque M, Tricoire-Leignel H, Nicotra A, Bourdoulous S, Turowski P, Male DK, Roux F, Greenwood J, Romero IA and Couraud PO. Blood-brain barrier-specific properties of a human adult brain endothelial cell line. Faseb J. 2005; 19(13):1872-1874.

6. Albuquerque RJ, Hayashi T, Cho WG, Kleinman ME, Dridi S, Takeda A, Baffi JZ, Yamada K, Kaneko H, Green MG, Chappell J, Wilting J, Weich HA, Yamagami S, Amano S, Mizuki N, et al. Alternatively spliced vascular endothelial growth factor receptor-2 is an essential endogenous inhibitor of lymphatic vessel growth. Nature medicine. 2009; 15(9):1023-1030.

7. Tran TC, Sneed B, Haider J, Blavo D, White A, Aiyejorun T, Baranowski TC, Rubinstein AL, Doan TN, Dingledine R and Sandberg EM. Automated, quantitative screening assay for antiangiogenic compounds using transgenic zebrafish. Cancer research. 2007; 67(23):11386-11392.

8. Uhlen M, Oksvold P, Fagerberg L, Lundberg E, Jonasson K, Forsberg M, Zwahlen M, Kampf C, Wester K, Hober S, Wernerus H, Bjorling L and Ponten F. Towards a knowledge-based Human Protein Atlas. Nature biotechnology. 2010; 28(12):1248-1250.

9. Thisse C, Thisse B, Schilling TF and Postlethwait JH. Structure of the zebrafish snail1 gene and its expression in wild-type, spadetail and no tail mutant embryos. Development (Cambridge, England). 1993; 119(4):1203-1215.

10. Pendeville H, Winandy M, Manfroid I, Nivelles O, Motte P, Pasque V, Peers B, Struman I, Martial JA and Voz ML. Zebrafish Sox7 and Sox18 function together to control arterial-venous identity. Developmental biology. 2008; 317(2):405-416.

11. Brown LA, Rodaway AR, Schilling TF, Jowett T, Ingham PW, Patient RK and Sharrocks AD. Insights into early vasculogenesis revealed by expression of the ETS-domain transcription factor Fli-1 in wild-type and mutant zebrafish embryos. Mechanisms of development. 2000; 90(2):237-252.

12. Jin SW, Beis D, Mitchell T, Chen JN and Stainier DY. Cellular and molecular analyses of vascular tube and lumen formation in zebrafish. Development (Cambridge, England). 2005; 132(23):5199-5209.
